# A genetic framework for proximal secondary vein branching in the *Arabidopsis thaliana* embryo

**DOI:** 10.1242/dev.200403

**Published:** 2022-06-27

**Authors:** Elizabeth Kastanaki, Noel Blanco-Touriñán, Alexis Sarazin, Alessandra Sturchler, Bojan Gujas, Francisco Vera-Sirera, Javier Agustí, Antia Rodriguez-Villalon

**Affiliations:** 1 Group of Plant Vascular Development, Swiss Federal Institute of Technology (ETH) Zurich, CH-8092 Zurich, Switzerland; 2 Group of RNA Biology, Swiss Federal Institute of Technology (ETH) Zurich, CH-8092 Zurich, Switzerland; 3 Instituto de Biología Molecular y Celular de Plantas, Consejo Superior de Investigaciones Científicas (CSIC)-Universitat Politècnica de València (UPV), 46022 Valencia, Spain

**Keywords:** Auxin canalization, Cell division, CLE peptides, Developmental plasticity, Embryogenesis, Vein patterning

## Abstract

Over time, plants have evolved flexible self-organizing patterning mechanisms to adapt tissue functionality for continuous organ growth. An example of this process is the multicellular organization of cells into a vascular network in foliar organs. An important, yet poorly understood component of this process is secondary vein branching, a mechanism employed to extend vascular tissues throughout the cotyledon surface. Here, we uncover two distinct branching mechanisms during embryogenesis by analyzing the discontinuous vein network of the double mutant *cotyledon vascular pattern 2* (*cvp2*) *cvp2-like 1* (*cvl1*). Similar to wild-type embryos, distal veins in *cvp2 cvl1* embryos arise from the bifurcation of cell files contained in the midvein, whereas proximal branching is absent in this mutant. Restoration of this process can be achieved by increasing *OCTOPUS* dosage as well as by silencing *RECEPTOR-LIKE PROTEIN KINASE 2* (*RPK2*) expression. Although *RPK2*-dependent rescue of *cvp2 cvl1* is auxin- and CLE peptide-independent, distal branching involves polar auxin transport and follows a distinct regulatory mechanism. Our work defines a genetic network that confers plasticity to *Arabidopsis* embryos to spatially adapt vascular tissues to organ growth.

## Abstract (precis)

**Summary:** A genetic framework involving *RPK2*, *CVP2*, *CVL1* and *OPS* reveals how *Arabidopsis* embryonic cotyledons modulate vascular branching for organ growth by an auxin-independent mechanism.

## INTRODUCTION

**Fig. 1.**
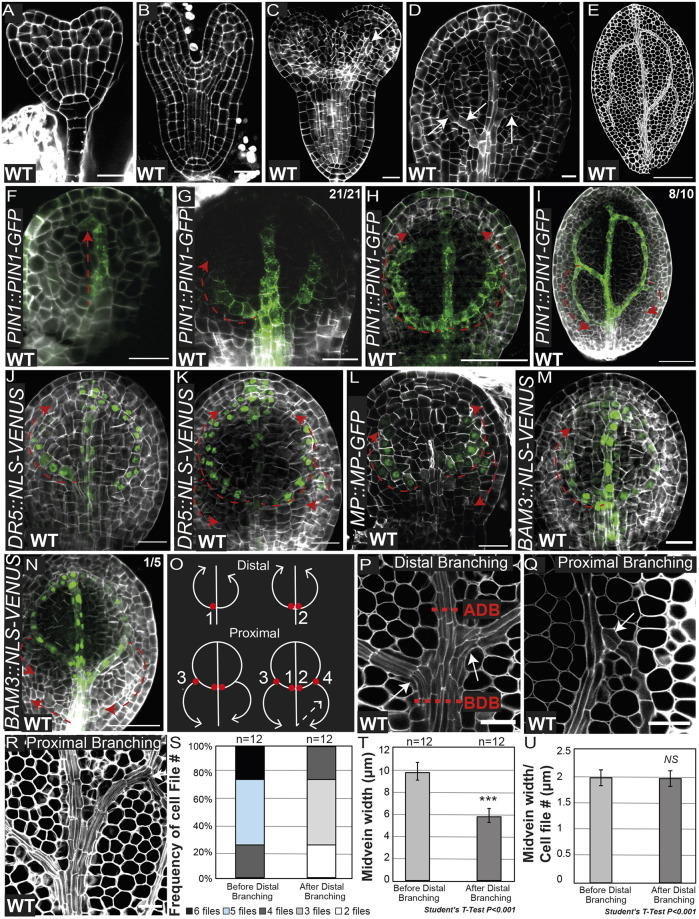
**Vein progression and branching in torpedo embryos.** (A,B) Representative pictures of mPS-PI-stained cell walls of embryos in the late transition stage (A) and late heart stage (B). (C,D) Analysis by confocal microscopy of extracted embryos from ovules and stained with the cell-wall dye SR2200. (C) Embryo in transition between the heart and early torpedo stages in which the future midvein is marked by a white arrow. (D) Cotyledon of early torpedo stage in which the secondary vein formation can be detected. Note that, in D, based on the elongated morphology of procambial cells, secondary vein formation is initiated from the midvein and progresses upwards towards the top of the cotyledon (marked by white arrows). (E) Cotyledon of a mature embryo in which the cotyledon vein network is completed. (F-I) Representative images of *PIN1::PIN1-GFP* early and late torpedo stage embryos stained with the cell-wall dye SR2200 showing the progression of midvein or primary vein (F), distal secondary vein (G,H) and proximal secondary vein (I) formation. Dashed red arrows represent the directionality of the forming veins. (J,K) Cotyledons from early torpedo stage embryos harboring *DR5::NLS-VENUS* showing the progression of distal secondary veins (J) as well as the initiation of the proximal secondary veins (K). (L-N) Early torpedo stage embryos harboring *MP::MP-GFP* (L) or *BAM3::NLS-3×VENUS* (M,N) showing cotyledon proximal vein formation occurring in a tip-to-base manner, except in N, in which proximal vein formation also proceeds in a base-to-tip manner. (O) Scheme representing the proposed branching sites of distal and proximal secondary veins. Distal branching points 1 and 2 are represented by the red dots and the direction of vein formation is represented by the white arrows. Proximal secondary vein branching, branching points 3 and 4 (red dots) and the direction of vein formation (white arrows) are represented in the lower panels. Note that distal versus proximal secondary vein formation occurs normally in opposing directions. The appearance of proximal veins in a base-to-tip manner is represented by a dashed white arrow. (P-R) Representative images of proximal and distal branching in embryonic cotyledons stained with mPS-PI and visualized by confocal microscopy. ADB, after distal branching; BDB, before distal branching. White arrows indicate proximal branching points. (S-U) Quantification of the frequency of appearance of the indicated number of cell files (S), average midvein width (T) and average midvein cell-file width (U) in the regions marked ADB and BDB in P. Note that the differences represented in T are not due to differences in the widths of procambial cells in these regions, as indicated in U. Data are shown as mean±s.d. NS, not significant; ***P<0.01 (two-tailed paired Student's *t*-test). Images are representative of two experiments. Scale bars: 20 µm (A-D,F,G,J-M,P-R); 50 µm (E,H,I,N).

The appearance of a continuous vascular network in plants, as a means for water and nutrient exchange among organs, greatly contributed to their conquering of a wide range of terrestrial ecosystems (Lucas et al., 2013; Agusti and Blazquez, 2020). The evolution of plants is characterized by the selection of a spatial arrangement of vascular strands (vascular patterns) that maximize their overall functionality (Lucas et al., 2013). In *Arabidopsis thaliana*, the vascular pattern of foliar organs is generated and maintained through the formation of continuous procambial cell files, which are further organized in vascular bundles comprising the conductive tissues phloem and xylem (Lavania et al., 2021). In most species, the patterning of leaf vascular tissues exhibits a high degree of plasticity (Scarpella, 2017); however, in *Arabidopsis* cotyledons, the robust and reproducible patterns of the vein network offer an ideal model to identify the positional and molecular cues underlying the development of the vein network. The vascular network in these organs includes a single primary vein (midvein), which extends along the central part of this organ and ensures the connection to the vascular system of the stem (Scarpella, 2017). As this organ expands laterally, a pair of secondary veins diverge from the midvein and extend toward the cotyledon margins, owing to the proliferation of the anticlinally dividing plate meristematic cells (Fig. 1O) (Scarpella, 2017; Tsukaya, 2021). These vascular cells are surrounded by mesophyll cells, which have been proposed to limit vein propagation in *Arabidopsis* leaves through their differentiation, thereby terminating the vein path (Scarpella et al., 2004). Similar to vascular cells, mesophyll cells derive from ground meristem (GM) cells located in the subepidermal layer of cotyledons (Lavania et al., 2021). During the specification of GM cells into either mesophyll or vascular cells, positional information determines the acquisition of cell identity (Sieburth, 1999; Scarpella et al., 2006). The widely accepted model for cotyledon/leaf vascular cell specification, the auxin canalization model, supports the idea that a directional auxin flow acts as a pre-pattern and reinforces vascular cell identity, delineating an incipient vascular path along the cells that were the most exposed to the auxin flow (Scarpella et al., 2006; Lavania et al., 2021). Indeed, specific subepidermal GM cells of the cotyledon will acquire a pre-procambial identity (Scarpella, 2017), which is associated with the expression of auxin-related genes such as *MONOPTEROS* (*MP*) or *ARABIDOPSIS THALIANA HOMEOBOX 8* (*ATHB8*)*.* The subsequent elongation of pre-procambial cells results in their transition to procambial cells (Lavania et al., 2021).

Although auxin is considered to be a necessary and sufficient signal for vein formation (Lavania et al., 2021), the molecular regulators underpinning the branching of vascular tissues have not been fully explored. Furthermore, the auxin canalization model itself cannot solely explain the appearance of an intermittent vascular network in vein mutants such as the double mutant *cotyledon vascular pattern 2* (*cvp2*) *cvp2-like 1* (*cvl1*) (Carland and Nelson, 2009). In particular, the simultaneous depletion of phosphatidylinositol 4,5-bisphosphate [PtdIns(4,5)P_2_] phosphatases CVP2 and its homolog CVL1 results in a reduction in vascular complexity and the appearance of off-path vascular islands (Carland and Nelson, 2009). Initially, CVP2 and CVL1 were suggested to generate phosphatidylinositol-4-phosphate (PtdIns4P), which is required to activate ARF-GAP SCARFACE (SFC) (Naramoto et al., 2009). Although the discontinuous vascular network observed in *sfc* mutants was found to be related to its role in controlling PIN1 endocytosis (Naramoto et al., 2009), further studies have demonstrated a wider range of subcellular activities that are modulated by CVP2 and CVL1, such as polarity establishment. In particular, augmented PtdIns(4,5)P_2_ levels enhanced subcellular trafficking towards the vacuole, a process that has been associated with the misspecification of root protophloem and companion cells (Gujas et al., 2020; Rodriguez-Villalon et al., 2015). The root vascular phenotypes associated with the skewed ratio of PtdIns4P and PtdIns(4,5)P_2_ can be rescued by introducing a *receptor-like protein kinase 2* (*rpk2*) mutation into a *cvp2 cvl1* background (Gujas et al., 2020). *RPK2* and its homolog *RECEPTOR-LIKE PROTEIN KINASE 1* (*RPK1*) were first described during embryogenesis to control protodermal identity, as evidenced by the expanded vascular domain found in *rpk1 rpk2* globular embryos (Nodine et al., 2007).

The morphological complexity of vascular tissues has thus far hindered our understanding of the sequence of events resulting in the establishment of the embryonic vein pattern. Here, we analyze the directionality of cotyledon vein formation during embryogenesis. We observed that two different mechanisms initiate the branching of distal secondary veins (the secondary veins located in the distal region of the cotyledon) and proximal secondary veins (the secondary veins that emerge from the distal veins in the proximal region of the cotyledon), and that the progression of both types of secondary veins exhibit opposite directionality. Whereas distal branching involves the bifurcation of the cell files comprising the midvein, proximal secondary veins arise from the branching of distal veins through an incompletely understood mechanism. Although distal branching involves PIN1 function, polar auxin transport does not appear to control vascular cell fate commitment, as revealed by the continuous vascular network observed in mutants lacking several PIN proteins (Verna et al., 2019). Furthermore, we report that both CVP2 and CVL1 are necessary to promote proximal branching, which is counteracted by the activity of RPK2. Our work demonstrates that silencing *RPK2* expression partially restores the cotyledon vein network complexity in *cvp2 cvl1* mutants by promoting vein branching, which is necessary to create proximal secondary veins. Through transcriptomic profiling and genetic assays, we found that a reduced activity of RPK2 restores the impaired cotyledon vein pattern in *cvp2 cvl1* embryos, in a manner independent of auxin and vascular-specific CLAVATA3/EMBRYO SURROUNDING REGION-RELATED (CLE) peptides. By expressing *RPK2* at the cotyledon margins, plants establish a vein network boundary beyond which veins do not extend. Additionally, we show that the positive vascular regulator *OCTOPUS* (*OPS*) promotes proximal branching*.* Taken together, our work supports a genetic network in which the positive regulation of vein branching by *CVP2*, *CVL1* and *OPS* is limited by *RPK2*, modulating the vein pattern to organ outgrowth and, in turn, maximizing the functionality of vascular tissues.

## RESULTS

### Two distinct branching mechanisms control cotyledon vein network formation in torpedo stage embryos

To gain insight into the contribution of procambial cell identity acquisition and cell proliferation in the cotyledon vein network, we first sought to characterize the cotyledon vascular ontogeny during embryogenesis. The emergence of procambial cell files can be detected by confocal microscopy based on their characteristic narrow and elongated morphology. Confocal microscopy analysis of Renaissance-stained embryos revealed the appearance of pre-procambial cell files at the early torpedo stage, in which a midvein was detected within the cotyledons (Fig. 1A-C). Although the complete establishment of the vein network was detectable in the late torpedo stage, elongated narrow cells diverging from the midvein were observed at earlier developmental time points (which we termed as the intermediate torpedo stage) (Fig. 1D,E). To further corroborate these observations, we monitored the auxin efflux PIN1 protein, the distribution of which is restricted to procambial and protodermal cells in torpedo embryos (Fig. 1F). We observed a progressive formation of the midvein that was concomitant with the base-to-tip appearance of distal secondary veins (Fig. 1F-I,O). The morphological progressive directionality of the distal secondary veins was corroborated through monitoring auxin response using *DR5::NLS-VENUS*, a widely used auxin response biosensor, and by gene expression analysis of *MP* (a pre/procambial marker) as well as the root phloem regulator *BARELY ANY MERISTEM 3* (*BAM3*) (Fig. 1J-O) (Scarpella et al., 2004; Przemeck et al., 1996; Rodriguez-Villalon et al., 2014)*.* Contrary to the base-to-tip growth of the distal secondary veins, PIN1 tagged with GFP polarly accumulates at the basal membrane of the vein network procambial cells (Fig. 1G,H), as previously reported (Scarpella et al., 2006). As the distal vascular strand extended in a base-to-tip manner, the polar accumulation of PIN1 at the basal membrane appeared to act as a mechanism to reinforce cellular identity in cells already committed to vascular cell fate. To assess the origin of the procambial cell files involved in the formation of distal secondary veins, we analyzed the region near the base of the cotyledon. Here, we observed that the number of cell files that constitutes the midvein was higher in the region where the distal veins had not yet branched out (Fig. 1P-S). In contrast, the numbers of cell files comprising the proximal and distal veins appeared to be similar (Fig. 1P-U). These observations indicate that distal secondary veins branch out directly from the cell files comprising the midvein, which can act as branching points to change their spatial progression. Instead, the branching of proximal veins does not appear to follow the same pattern, as the number of cell files in the regions adjacent to the branching points remained similar.

### GM cells fail to commit to procambial cell identity in *cvp2 cvl1* mutants

**Fig. 2.**
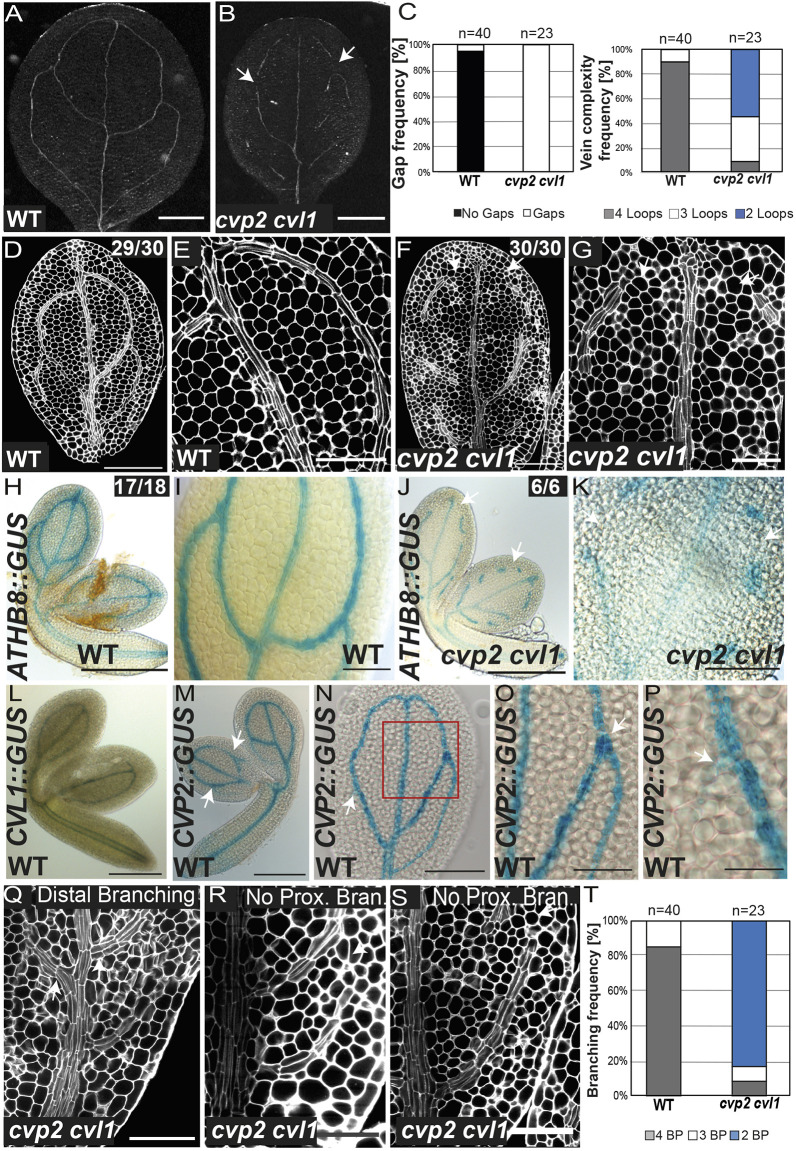
**Cotyledon vein defects of *cvp2 cvl1*.** (A,B) Cleared 7-day-old cotyledons from WT and *cvp2 cvl1* mutants imaged with a stereomicroscope in bright field on a black background. White arrows indicate vein gaps. (C) Quantification of the frequency of ground meristem cells surrounding a disconnected vascular island (gap frequency) and vein complexity in WT and *cvp2 cvl1* cotyledons. *n*=23-40 for each genotype. This quantification is part of the experiment represented in Fig. 5E,F. (D-G) Confocal microscopy analysis of the vein pattern of mature embryonic cotyledons (vein network at its complete stage) of WT and *cvp2 cvl1* having undergone mPS-PI staining. E and G are magnifications of D and F, respectively. White arrows indicate vein gaps. Note that the picture shown in D is the same as the one that appears in Fig. 1E. (H-K) *ATHB8::GUS* expression in WT and *cvp2 cvl1* mature embryonic cotyledons. I and K are magnifications of the veins displayed in H and J, respectively. White arrows indicate vein gaps. (L-P) Expression pattern of *CVL1::GUS* and *CVP2::GUS* in embryos. Magnification of an embryonic cotyledon (N) displaying *CVP2* expression in the proximal branching points is shown in O and P. White arrows indicate branching points. (Q-S) mPSI-PI staining of mature embryonic cotyledons of WT and *cvp2 cvl1* showing distal versus proximal branching. White arrows indicate the presence or absence of branching points. Note the two phenotypes observed in *cvp2 cvl1* mutants when there is a third branching event (R) or not (S). (T) Quantification of the frequency of the reduced vein complexity observed in each genotype. *n*=23-40. BP, branching points, counted as the initiation (even if not completed) of a new secondary vein. Images are representative of two independent experiments. Scale bars: 500 µm (A,B); 200 µm (H,J,L,M); 100 µm (N); 50 µm (D,F,I,K,O,P,Q-S); 20 µm (E,G).

To better understand the spatiotemporal arrangement of vascular formation in cotyledons during embryogenesis, we decided to exploit the discontinuous cotyledon venation pattern in *cvp2 cvl1* mutants as a model system. Consistent with previous studies (Carland and Nelson, 2009), 7-day-old *cvp2 cvl1* cotyledons exhibited a discontinuous and more simplified cotyledon vein network compared with wild-type (WT) cotyledons (Fig. 2A-C). However, the midvein was always morphologically intact, as visualized in cleared cotyledons (Fig. 2B). These vascular defects originated during embryogenesis, as observed in cotyledons in mature embryos stained with modified pseudo-Schiff propidium iodide (mPS-PI) (Fig. 2D-G). At this developmental stage, procambial cells were observed as elongated, narrow elements, whereas the surrounding mesophyll cells were spherical (Fig. 2E,G). The latter morphology was also detected in between vascular islands, indicating that GM cells in *cvp2 cvl1* mutants failed to commit to procambial cell identity (Fig. 2G) and became mesophyll cells instead. This notion was corroborated by the lack of *ATHB8* expression in the spherical cells flanking *cvp2 cvl1* vascular islands, whereas a continuous expression of this gene within the cotyledon vein network was detected in WT embryos (Fig. 2H-K). As *CVP2* and *CVL1* were expressed in the vein domain from the globular stage (Fig. S2; Fig. 2L-P), we next evaluated the vascular identity domains in early embryonic stages in *cvp2 cvl1* mutants. In globular embryos, the expression of *SHORT ROOT* (*SHR*) or *MP* marks the onset of vascular formation and delineates the separation of the future ground tissues (De Rybel et al., 2016; Scarpella, 2017). At this particular stage, none of the vascular markers that were analyzed showed aberrant expression in *cvp2 cvl1* embryos, with the exception of *MP* in globular embryos, which showed very weak expression (Fig. S2F-M). Together, these results indicate that the cotyledon vein network discontinuities in *cvp2 cvl1* mutants are not due to impaired vascular identity domains during the early stages of embryogenesis and must occur in later embryonic developmental stages.

### CVP2 and CVL1 activities are required to modulate cell division and proximal branching

Matching the embryonic stages relevant to their expression, we observed a plethora of aberrant morphologies in *cvp2 cvl1* double-mutant embryos (Fig. S3). Whereas some mutant embryos resembled WT embryos (phenotype A), several globular embryos exhibited an excessive number of divisions in the suspensor and hypophysis (phenotype B, Fig. S3). Moreover, cells in the globular embryos of *cvp2 cvl1* mutants displayed aberrations in the number and the orientation of their divisions (Fig. S3). We observed a high embryo abortion rate in *cvp2 cvl1* siliques (∼50%), which we believe was partially contributed by the aberrant divisions observed primarily in the hypophysis, given that abnormal suspensor development has been reported to be lethal (ten Hove et al., 2015). Although subsequent developmental stages appeared to be morphologically similar to WT, we cannot exclude the appearance of mild division defects occurring within the vascular domain due to the technical limitations of working with torpedo stage embryos (Fig. S3). However, we observed that proximal branching was absent in the cotyledon vein network of *cvp2 cvl1* embryos (Fig. 2Q-T), consistent with *CVP2* expression at the branching points (BPs) (Fig. 2N-P). In the event of secondary vein formation within the proximal cotyledon region, these procambial cell files appeared to be connected to the basal end of the midvein, arising from the cells that form the midvein (Fig. 2Q,R, marked by a white arrow). Indeed, these incipient cell files propagated in a base-to-tip manner (Fig. 2S). Taken together, the presence of an intact midvein and the lack of proximal secondary vein branching imply that the activities of CVP2 and CVL1 are required to initiate proximal secondary veins at the proximal branching point.

### Polar distribution of PIN1 is not altered in *cvp2 cvl1* mutants

**Fig. 3.**
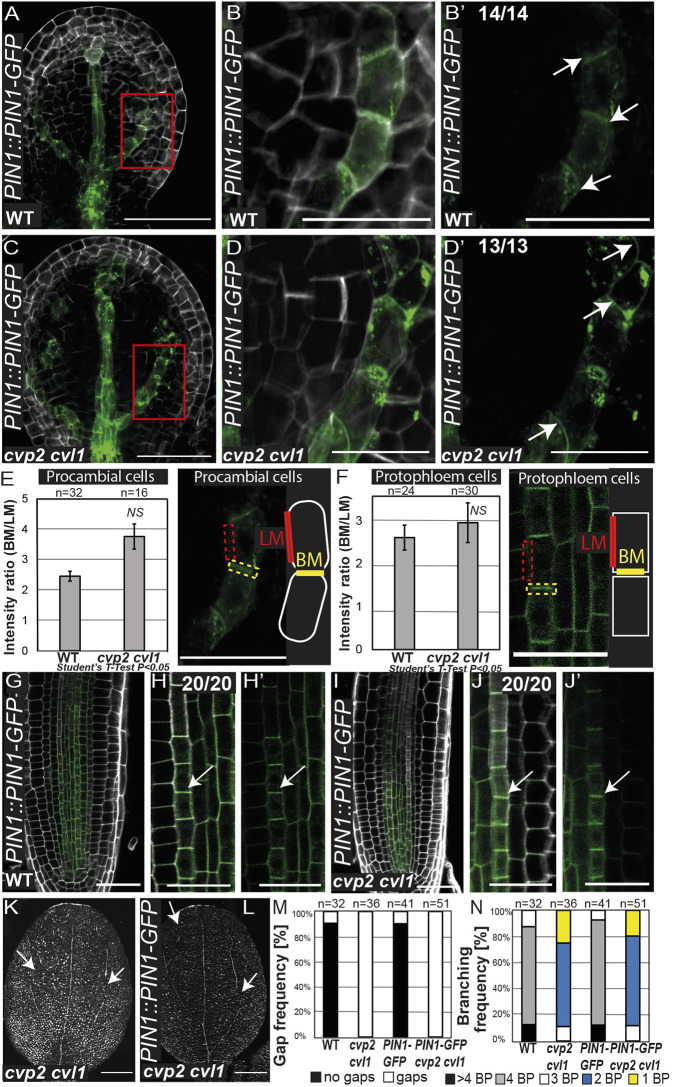
**Polar distribution of PIN1 is not affected in *cvp2 cvl1* mutants.** (A-D′) Confocal microscopy analysis of early torpedo stage embryos of the indicated genotypes stained with SR2200 showing PIN1-GFP distribution in distal secondary veins as they progressively form upwards. Magnifications of the region in red squares in A and C are shown in B,B′ and in D,D′, respectively. White arrows indicate the polar distribution of PIN1 in the cell. (E,F) Quantification of the polar distribution of PIN1-GFP in procambial and protophloem cells as a ratio of the GFP signal detected in the basal membrane (BM) versus the lateral membrane (LM). Data are shown as mean±s.d. NS, not significant (two-tailed paired Student's *t*-test). (G-J′) 6-day-old roots harboring *PIN1::PIN1-GFP* in WT and *cvp2 cvl1* backgrounds showing PIN1 localization and strong basal polarization in the protophloem strand. Magnification of differentiating cells in the protophloem in WT (G) and *cvp2 cvl1* (I) are shown in H,H′ and J,J′, respectively. White arrows indicate the polar distribution of PIN1 in the cell. (K,L) Representative images of 7-day-old cleared cotyledons of the indicated genotypes. White arrows indicate vein gaps. (M,N) Quantification of gap frequency (M) and branching (N) in the indicated genotypes. *n*=32-51 for each genotype. BP, branching points. Images are representative of two experiments. Scale bars: 50 µm (A-D′,G,I); 20 µm (H,H′,J,J′); 200 µm (K,L).

**Fig. 4.**
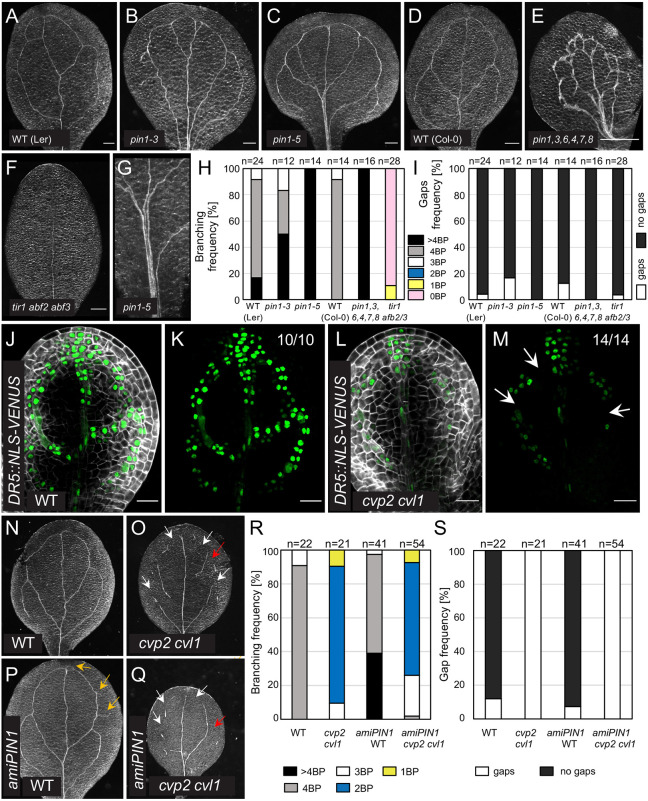
**PIN-mediated auxin transport modulates distal branching but not vein continuity in embryonic cotyledons.** (A-G) Representative images of 7-day-old cleared cotyledons of the indicated genotypes. Note that *pin1* single mutants are in a Ler background. Magnification of the midvein region where distal branching occurs in *pin1-5* (C) is displayed in G. (H,I) Quantification of branching (H) and gap (vascular discontinuities) (I) frequency in the indicated genotypes. *n*=12-28 for each genotype. BP, branching points, counted as the initiation (even if not completed) of a new secondary vein. (J-M) Auxin distribution analyzed by *DR5* expression in WT (J,K) and *cvp2 cvl1* (L,M) embryonic cotyledons counterstained with SR2200. K and M display only the GFP signal. White arrows indicate the absence of the marker in the vein gaps. (N-Q) Representative images of 7-day-old cleared cotyledons of the indicated genotypes. Orange arrows mark extra branching points, red arrows indicate the absence of proximal branching formed in a tip-to-base manner and white arrows mark gaps. (R-S) Quantification of branching (R) and gap (S) frequencies in the indicated genotypes. *n*=21-54 for each genotype. BP, branching points, counted as the initiation (even if not completed) of a new secondary vein. Images are representative of two experiments. Scale bars: 200 µm (A-F); 20 µm (J-M).

To gain further insight into the vein network branching defects observed in *cvp2 cvl1* embryonic cotyledons, we first assessed the role of auxin and its PIN1-mediated transport. Confocal microscopy analysis of PIN1-GFP in *cvp2 cvl1* torpedo embryos showed a normal basal polarization of the auxin carrier in the distal secondary veins (Fig. 3A-D′), which we confirmed by quantifying PIN1 accumulation in the basal membrane compared with its accumulation in a lateral membrane (Fig. 3E). Nonetheless, we could observe PIN1 aggregates in the intracellular space of *cvp2 cvl1* embryos, likely due to the enhanced trafficking from the plasma membrane to vacuoles, which has previously been associated with this genetic background (Gujas et al., 2017). Similarly, in root protophloem cells, where PIN1 polarly accumulated at the basal membrane, we did not observe any abnormal distribution of this auxin efflux carrier (Fig. 3F-J′). Previous reports have suggested that PIN1 activation requires the function of PIP5K1 and PIP5K2 (Marhava et al., 2020), which catalyze the inverse enzymatic reaction to that of CVP2 and CVL1 (Gujas and Rodriguez-Villalon, 2016). To determine the extent to which the vascular phenotypes observed in *cvp2 cvl1* cotyledons may be due to a perturbed PIN1 activity, we analyzed the vascular phenotypes of *pin1* seedlings. A plethora of cotyledon defects have been described in distinct *pin1* mutants, including fused cotyledons and aberrant morphologies (Friml et al., 2003). To overcome the impact of defective organogenesis in the analysis of vascular patterning, we focused on separated cotyledons from the loss-of-function mutants *pin1-3* and *pin1-5*. We consistently observed increased distal branching in *pin1* single mutants, whereas the continuity of the vascular strands appeared to be intact (Fig. 4A-C,G-I). Likewise, the simultaneous depletion of other vascular PIN carriers, such as *PIN3*, *PIN4*, *PIN6*, *PIN7* and *PIN8* (*pin1*,*3*,*4*,*6*,*7*,*8* mutant), did not result in a discontinuous vein pattern (Fig. 4D,E,H,I), although the duplication of the midvein and subsequent bifurcation in distal veins was seen in these mutants (Fig. 4E). These observations indicated that the polar auxin transport represses distal branching. An increase in PIN1 dosage by introducing *PIN1::PIN1-GFP* (Yanagisawa et al., 2021) (Fig. S1A) in *cvp2 cvl1* did not aggravate the vascular phenotype of the double mutant (Fig. 3K-N). Moreover, the introduction of an artificial microRNA (ami) against *PIN1* (*amiPIN1*) in *cvp2 cvl1* plants restored neither the continuous vein network nor the proximal branching defects observed in *cvp2 cvl1* embryos (Fig. 4N-S), despite the efficacy of the *amiPIN1* construct to reduce *PIN1* expression levels (Fig. S1B). These observations demonstrated that *cvp2 cvl1* defects in proximal branching are independent of PIN1 activity. To identify the origin of vascular identity failure observed in *cvp2 cvl1* embryonic cotyledons, we analyzed auxin response. The deficient activity of the auxin receptors TRANSPORT INHIBITOR RESPONSE 1/AUXIN SIGNALING F-BOX (TIR1/AFB) suppresses the formation of secondary veins whereas the midvein appears still intact (Fig. 4F), a phenotype consistent with previous publications (Mazur et al., 2020). To further investigate whether the inability of *cvp2 cvl1* cells to perceive auxin could explain the appearance of a discontinuous vascular network in this mutant, we monitored the distribution of the auxin response biosensor *DR5::NLS-VENUS*. Confocal microscopy analysis of embryonic cotyledons showed a positive correlation between cells exhibiting vascular morphology and *DR5* expression (Fig. 4J-M). However, no signal was detected in the cells flanking vascular islands (Fig. 4L,M). Taken together, our observations suggest that, although polar auxin transport modulates distal branching, it is not responsible for the vein discontinuities and proximal branching defects observed in *cvp2 cvl1* embryos.

### Silencing of *RPK2* rescues the proximal branching defects of *cvp2 cvl1* embryonic cotyledons

**Fig. 5.**
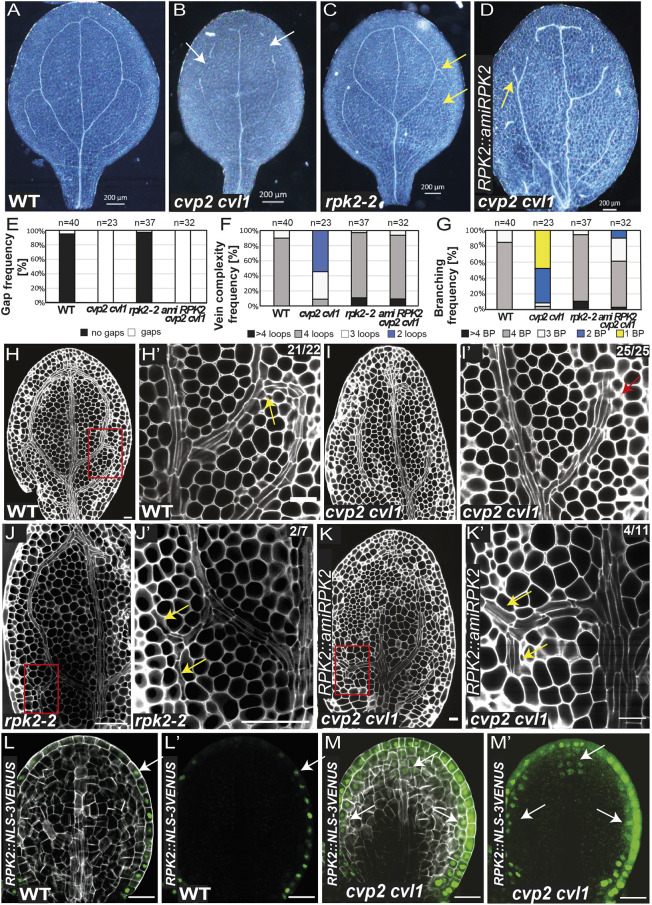
**Silencing of *RPK2* expression rescues the branching defects of *cvp2 cvl1*.** (A-D) Analysis of the continuity and complexity of the cotyledon vein network in 7-day-old seedlings from the indicated genetic backgrounds. White arrows indicate vein gaps, and yellow arrows indicate proximal branching. (E-G) Quantification of gap (E), vein complexity (F) and branching (G) frequencies observed in the cotyledons of the plants shown in A-D. *n*=23-50 for each genotype. BP, branching points. (H-K′) mPS-PI-stained embryos displaying the vein pattern of the indicated genotypes. H′,I′,J′ and K′ show magnified views of the squared regions in H,I,J and K, respectively. Yellow arrows mark proximal branching and the red arrow marks the lack of proximal branching. (L-M′) Confocal microscopy analysis of *RPK2* expression in the cotyledons of torpedo embryos from the indicated genotypes stained with SR2200. L′ and M′ show only the GFP signal. White arrows indicate the localization of *RPK2::NLS-3×VENUS* in the cell. Images are representative of two experiments. Scale bars: 200 µm (A-D); 50 µm (J,J′); 20 µm (H-I′,K,K′,L-M′).

Considering that auxin transport itself cannot explain the vascular defects observed in *cvp2 cvl1* mutants, we explored the cotyledon vascular ontogeny in plants with deficient activity of RPK2. We have previously reported that silencing of *RPK2* in *cvp2 cvl1* (using *amiRPK2*) restores the continuity of the root protophloem strands in this mutant (Gujas et al., 2020). Notably, a partial silencing of *RPK2* did not rescue the discontinuous secondary veins of *cvp2 cvl1* (Fig. 5A,B,D,E). Instead, an increased number of branching points was observed in these lines (Fig. 5A,B,D-G,K,K′). In light of these results, we decided to further elucidate the potential role of RPK2 in cotyledon vein patterning. Examination of the vascular pattern in *rpk2* mutants revealed the occasional appearance of additional proximal branching points within the embryonic cotyledon vein network, even though a complete/closed additional aerole was rarely observed (Fig. 5C,F,G). Furthermore, divisions giving rise to a bifucarted vein path could also be occasionally detected in mPS-PI-stained *rpk2* embryos (Fig. 5H-K′). Consistent with previous studies (Nodine et al., 2007), *RPK2* expression was mainly detected in the protoderm and in some cells of the adjacent cell file (Fig. 5L,L′). Confocal microscopy analysis of *RPK2* expression in *cvp2 cvl1* mutants, however, revealed a slightly broader expression pattern towards the inner cell layers, which was strongest at the tip of the cotyledon (Fig. 5M,M′). The expression of *RPK2* in the vein-free cotyledon margins suggests that *RPK2* may repress vein branching in a non-cell-autonomous manner.

### The partial *RPK2-*mediated restoration of the *cvp2 cvl1* vascular phenotype is PIN1-independent

**Fig. 6.**
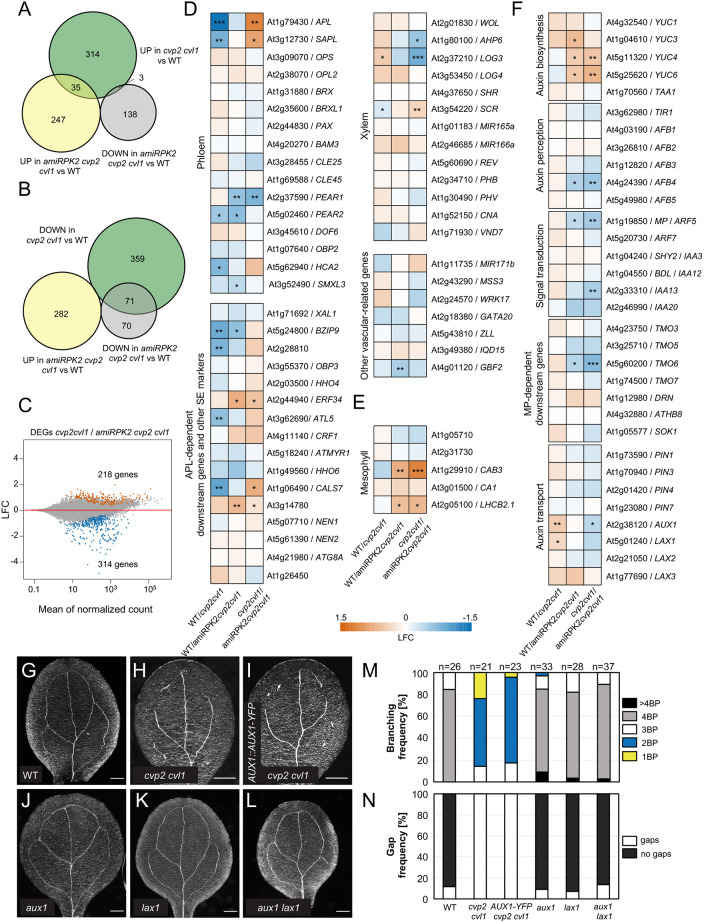
**Differential gene expression analysis between WT, *cvp2 cvl1* and *amiRPK2 cvp2 cvl1* mutants.** (A,B) Venn diagrams showing the overlap of upregulated (A) and downregulated (B) genes in *cvp2 cvl1* with the DEGs in *amiRPK2 cvp2 cvl1*. (C) MA plot showing the log_2_ fold change (LFC) of each gene over the mean of normalized counts. (D-F) Heatmaps show enrichment (LFC) of genes with known roles in vascular development (D), with known expression in mesophyll cells (E) and involvement in auxin biosynthesis, signaling and transport (F). *P<0.05; **P<0.01; ***P<0.001 (DEseq2 analysis). (G-L) Bright-field images of 7-day-old cotyledons of the indicated genotypes. Images are representative of two experiments. Scale bars: 200 μm. (M,N) Quantification of branching (M) and gap (N) frequencies of the vein network phenotypes observed in the cotyledons represented in G-L. *n*=21-37 for each genotype. BP, branching points, counted as the initiation (even if not completed) of a new secondary vein.

To gain further insight into the mechanisms by which RPK2 modulates vein patterning, we monitored the transcript profiles of WT, *cvp2 cvl1* and *amiRPK2 cvp2 cvl1* embryos between the early torpedo and bent cotyledon stages. Both mutants showed hundreds of differentially expressed genes (DEGs) compared with WT (782 for *cvp2 cvl1*, 423 for *amiRPK2 cvp2 cvl1*). Remarkably, silencing of *RPK2* resulted in a partial complementation at the transcriptomic level of the *cvp2 cvl1* defects (Fig. 6A-C), evident by, for example, the repression of the augmented transcript levels of *ALTERED PHLOEM* (*APL*) (Bonke et al., 2003) and *SISTER OF APL* (*SAPL*) (Ross-Elliott et al., 2017) of *cvp2 cvl1* embryos (Fig. 6D). Consistent with this result, the induced expression of *AT2G28810* (Furuta et al., 2014) or the callose biosynthetic enzyme *CALS7* (Vaten et al., 2011) *–* known targets of *APL –* was reverted to WT levels in *amiRPK2 cvp2 cvl1* mutants (Fig. 6D). Moreover, the expression of genes associated with mesophyll identity, such as *CHLOROPHYLL A/B BINDING PROTEIN 3* (*CAB3*) (Mitra et al., 1989), was not affected in *cvp2 cvl1* embryos (Fig. 6E), arguing against an altered genetic program of mesophyll cells as responsible for the misspecification of *cvp2 cvl1* procambial cells. Surprisingly, our transcriptomic analysis did not reveal altered expression levels in either auxin biosynthetic genes or auxin-related genes in *cvp2 cvl1* embryos (Fig. 6F), with the exception of the *AUXIN TRANSPORTER 1* (*AUX1*) and *AUXIN TRANSPORTER-LIKE PROTEIN 1* (*LAX1*) (Swarup and Bhosale, 2019). Contrary to *cvp2 cvl1* mutants, cotyledons of *aux1* and *lax1* null mutants exhibited a continuous vein network, even when both genes were simultaneously knocked out (Fig. 6G,J-N). Introgression of the *AUX1-GFP* transgene in *cvp2 cvl1* did not rescue the vascular phenotype of this mutant (Fig. 6G-I,M,N, Fig. 1A), suggesting that the *rpk2-*mediated rescue of *cvp2 cvl1* was not directly due to the modulation of auxin influx and its biosynthetic pathways. To exclude a potential regulation of PIN1-mediated auxin distribution by RPK2 as the restoring mechanism for *cvp2 cvl1* defects, we performed chemical treatments using 1-naphtylphathalamic acid (NPA), a widely used auxin transport inhibitor in leaves. These organs form *de novo* during the post-embryonic growth of the plant. The establishment of the vascular strands in leaves is believed to be regulated in a similar way to cotyledons, even if the directionality of the secondary strands differs (Lavania et al., 2021). Under NPA treatment, the acropetal transport of auxin that directs the growth of the midvein has been shown to be altered, resulting in the duplicated formation of midveins (Scarpella et al., 2006). Additionally, secondary vein formation is slightly affected, whereas tertiary vein formation may appear to be suppressed (Scarpella et al., 2006). Similarly to WT and *cvp2 cvl1* plants, NPA-treated *rpk2* leaves appeared to be affected in terms of vein complexity (Fig. S4A,F). Moreover, root growth inhibition of *rpk2* roots, which were subjected to NPA treatments, was very similar to that observed in WT and *cvp2 cvl1* plants (Fig. S4L). These observations imply that RPK2 activity is not necessary to integrate auxin polar transport cues into the establishment of the vein pattern, despite the rescue of the *cvp2 cvl1* leaf vein pattern through silencing of *RPK2* (Fig. S4G-I). Consistent with these results, live-cell image analysis of PIN1-GFP localization in *rpk2* mutants did not reveal any defective PIN1 localization in this genetic background. Given the reduced offspring of *rpk2*, we decided to assess PIN1 polarity within the root stele. Within the stele, PIN1 polarly accumulated at the basal cell membrane. The polar PIN1 distribution in *rpk2* roots (Fig. S4J-K′), taken together with the unaltered response of this mutant to NPA treatments, suggests that RPK2 activity in modulating vein emergence is independent of PIN1-mediated auxin transport.

### RPK2-mediated suppression of proximal branching is independent of CLE peptides

To explore further the underlying molecular mechanisms of RPK2-mediated repression of secondary proximal veins, we analyzed the potential involvement of CLE peptides in controlling this process. The *rpk2* loss-of-function mutant is resistant to the root growth-inhibition effect of a vast array of CLE peptides, including CLAVATA 3 (CLV3) and CLE45 (Gujas et al., 2020). In particular, RPK2 has been shown to perceive the CLV3 peptide, thereby negatively regulating root and shoot apical meristem cell proliferation (Racolta et al., 2018). Moreover, we have previously shown that the *rpk2*-mediated restoration of a continuous root protophloem strand in *cvp2 cvl1* mutants involves CLE45 (Gujas et al., 2020). To investigate the potential role of both peptides in orchestrating vascular patterning in cotyledons during embryogenesis, we cleared the cotyledons from *clv3* and *cle45* mutant seedlings (Yamaguchi et al., 2017)*.* Neither *cle45* nor *clv3* cotyledons showed any perturbation in their vein pattern or vein network complexity (Fig. S5A-D,G), suggesting either that these peptides are not involved in regulating cotyledon vein networks or that there is a high redundancy within this family of peptides. An overexpression of *CLE45* has been shown to result in lethality (Depuydt et al., 2013). Thus, we aimed at increasing *CLE45* and *CLV3* expression specifically during embryogenesis. To this end, we generated transgenic lines expressing *CLE45* and *CLV3* under the *ABSCISIC ACID INSENSITIVE 3* (*ABI3*) promoter. *ABI3* is broadly expressed in the embryo, with expression starting at the globular stage and lasting until embryo maturation, and ceases to be expressed after germination (To et al., 2006). Consistent with the previous results (Fig. S5), analysis of 8-day-old *ABI3::CLE45* seedlings did not reveal any vascular defects in the cotyledons, implying that RPK2-mediated regulation of vascular patterning is independent of CLE45 (Fig. S5E,G)*.* In contrast, a discontinuous vascular network in 25% of *ABI3::CLV3* seedlings was detected, compared with a frequency of 5% in WT plants (Fig. S5F,G). This feature was associated with reduced proximal branching and, in turn, vascular complexity (Fig. S5F,G). Our results indicated that, despite this peptide having the ability to suppress proximal vein branching, its restricted expression at the shoot apical meristem (Brand et al., 2002) likely excludes it as the ligand responsible for RPK2-mediated control of proximal branching.

### OPS promotes proximal branching in embryonic cotyledons

**Fig. 7.**
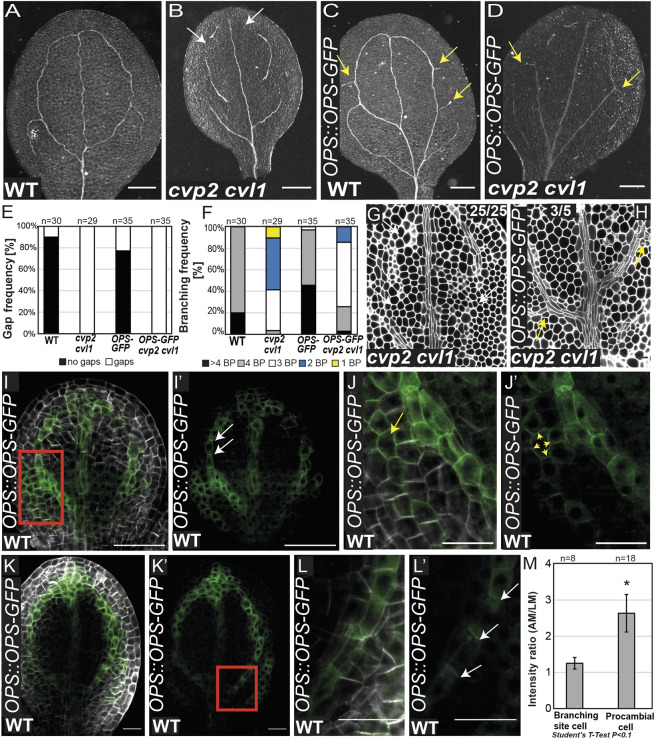
**OPS promotes proximal branching in WT and *cvp2 cvl1* embryonic cotyledons.** (A-D) Representative images of cleared 8-day-old cotyledons from the indicated genotypes imaged with a stereomicroscope in bright field on a black background. White arrows mark vein gaps and yellow arrows indicate additional proximal branching sites. (E,F) Quantification of gap and branching frequency of the vein network phenotypes observed in the cotyledons represented in A-D. *n*=29-35 for each genotype. BP, branching points, counted as the initiation (even if not completed) of a new secondary vein. (G,H) mPS-PI-stained embryos visualized by confocal microscopy for the indicated genotypes. White arrows (G) indicate vein gaps, yellow arrows (H) indicate proximal branching points. (I-L′) Visualization of OPS distribution in early (I-J′) and late (K-L′) torpedo stage embryos stained with SR2200. J and L show magnified views of the branching regions shown in I and K, respectively. In I′,J′,K′ and L′, only the GFP signal is shown. White and yellow arrows show polar and non-polar distributions of OPS in the cell, respectively. (M) Quantification of OPS polarity in cells from early and late torpedo stages as the mean±s.d. of the ratio of the GFP signal between the apical membrane (AM) and lateral membrane (LM). *P<0.1 (two-tailed paired Student's *t*-test). Scale bars: 200 μm (A-D); 20 µm (G,H); 50 µm (I,I′,K,K′); 20 µm (J,J′,L,L′).

Another factor involved in the regulation of procambial cell division in embryonic cotyledons is the plasma membrane-associated protein OPS (Truernit et al., 2012). In *ops* embryos, a reduced number of cell files in distal secondary veins can be detected as the procambial cells fail to periclinally divide (Roschzttardtz et al., 2014) (Fig. S6A). Live-cell imaging analysis of OPS distribution revealed a broader expression domain than that of the future vein path in intermediate torpedo stage embryos (Fig. 7I,I′) and a non-polar cellular distribution in cells not within the vein path (Fig. 7J,J′). In contrast, OPS was polarly distributed in the apical cell membrane of procambial cells in intermediate torpedo stage embryos and in mature embryos, once the vein network had achieved its final complexity (Fig. 7K-L′,M). OPS distribution was also detected in the non-procambial cells that were adjacent to the branching point and close to the cotyledon margin of intermediate torpedo embryos, near the *RPK2* expression domain (Fig. 7I-J). Interestingly, an increase in OPS protein dosage by introducing the hyperactive GFP-tagged OPS protein (Fig. S1A) (Breda et al., 2017) increased the number of proximal branching points in WT plants (Fig. 7A,C,F). Likewise, an increase in branching points as well as an overall more continuous cotyledon vein network was observed in *cvp2 cvl1* plants, even though vein pattern discontinuities persisted (Fig. 7A-H). Consistent with these findings, the mild proximal branching phenotype observed in *cvp2* single mutants was more pronounced when introgressed in the *ops* genetic background (Fig. S6A-C), mimicking *cvp2 cvl1*. These results confirmed that CVP2 and CVL1 are necessary to establish the proximal branching of secondary veins, a process enhanced by the positive vascular regulator OPS and counteracted by RPK2.

## DISCUSSION

### CVP2 and CVL1 regulate the specification of provascular cells and proximal branching

Within a multicellular organ, the self-establishment of tissue patterns such as vascular tissues requires the coordinated activity of oriented cell divisions and cell fate commitment (Lavania et al., 2021). Our data reveal that *CVP2* and *CVL1* contribute to the regulation of vein patterning by modulating vascular specification and the branching points of proximal secondary veins (Fig. 2). On the one hand, *cvp2 cvl1* cotyledons exhibit a discontinuous vein network because of GM cell misspecification into mesophyll instead of provascular cells (Fig. 2). A defective vascular identity matches with the lack of auxin activity in these cells, as revealed by auxin biosensors (Fig. 4L,M). However, the lack of this hormone in these cells does not correlate with a significantly different auxin transcriptional response in *cvp2 cvl1* embryos at the earlier torpedo stage of development (Fig. 6). Moreover, our analysis of vascular identity domains in *cvp2 cvl1* globular and heart stage embryos did not reveal defects in the establishment of vascular versus ground tissue domains during embryogenesis (Fig. S2). Thus, an alternative explanation consistent with the *cvp2 cvl1* phenotypes is that a premature differentiation of mesophyll cells terminates vein propagation, a phenomenon that has been described previously in leaves (Scarpella et al., 2004). However, further experiments are required to elucidate whether *CVP2* and *CVL1* participate in repressing mesophyll differentiation, even if the transcripts associated with mesophyll identity did not appear to be significantly altered in *cvp2 cvl1* embryos, as evidenced by our transcriptomic profiling (Fig. 6E). Alternatively, the deficient phloem-mediated sugar transport in *cvp2 cvl1* cotyledons (Rodriguez-Villalon et al., 2015; Carland and Nelson, 2009) may inhibit the regulation of GM specification into procambial cells. Further studies are required to assess whether sucrose intervenes in the conversion of GM into either mesophyll or procambial cells and whether a perturbed sucrose distribution translates to the appearance of vascular islands in *cvp2 cvl1* mutants. Although these disconnected vascular islands were frequently observed in the *cvp2 cvl1* cotyledon vein network, the midvein always appeared to be intact (Fig. 2). In cotyledons, the midvein originates directly from a pool of cells located in the vicinity of the shoot apical meristem, which start to elongate and divide parallel to the proximo-distal axis of cotyledon growth at the torpedo stage (Nelson and Dengler, 1997). This process is mostly regulated by auxin signaling factors such as *ARF6/CULLIN1* or *ARF5/MP* (Scarpella et al., 2004, 2006), implying that the activity of these factors remains unaffected by the loss of *CVP2* and *CVL1* during embryogenesis until the early torpedo stage. Several vascular-associated genes appeared to be mis-expressed in *cvp2 cvl1* embryos, even though *RPK2* silencing only reverted *APL* (and downstream target genes of *APL*) and *SAPL* expression (Fig. 6). On the other hand, the reduced vein network complexity described in *cvp2 cvl1* cotyledons reflects the inability of proximal secondary veins to initiate branching. Taken together with the aberrant divisions detected in the suspensor and hypophysis (Fig. S3), these observations suggest that the activity of both CVP2 and CVL1 may be required to a certain extent to control the orientation of cell divisions. Interestingly, closer examination of the branching points in WT plants revealed a periclinal division of a distal vein cell next to another cell harboring either a vascular marker or *DR5* (Fig. S7). Although it appears possible that a periclinal division precedes the formation of vascular cells from which the incipient proximal secondary vein will extend, we cannot exclude the possibility at this stage that a plate meristematic cell adjacent to the vascular cells is recruited at the branching point to give rise to the new vein. Another interesting aspect of this process is whether auxin is involved in the coordination of proximal vein branching. Inhibition of polar auxin transport by depleting the activity of PIN transporters or silencing their expression resulted in an elevated number of distal veins with additional distal branching points (Fig. 4). Although these results indicated that polar auxin cues repress distal branching, they cannot explain the reduced cotyledon vein network complexity or the intermittent cotyledon vein patterns that were observed in *cvp2 cvl1* plants. Our observations show that PIN1 polarity was unaltered in *cvp2 cvl1* veins (Fig. 3). Additionally, alteration of PIN1 dosage did not alter the branching defects observed in *cvp2 cvl1* mutants (Figs 3 and 4), indicating that at least another mechanism independent of PIN1 must be responsible for this phenotype. Interestingly, recent studies have shown that the reduced leaf vein complexity of auxin biosynthetic mutants can be partially restored upon inhibition of auxin transport (Kneuper et al., 2021). Local auxin biosynthesis thus plays a key role in determining the paths of future veins in leaves (Kneuper et al., 2021). Moreover, the expression of auxin biosynthetic enzymes or *PIN1* is independent of polar auxin transport (PAT) (Kneuper et al., 2021). As auxin response in *cvp2 cvl1* cotyledons as seen by *DR5* distribution was discontinuous in the vein path, it is plausible that this reflects a perturbed auxin biosynthetic pathway in certain embryonic cells. Although our transcriptomic analysis did not reveal any changes in the main auxin biosynthetic enzymes (Fig. 6), further single-cell analysis as well as enzymatic activity assays will be required to precisely assess the potential role of local auxin production in vascular cell fate acquisition in *cvp2 cvl1* cotyledons. Alternatively, it is possible that an auxin-independent mechanism preceding *PIN1* expression is required to modulate the acquisition of vascular cell identity and, in turn, the future vein path.

### *RPK2* constrains proximal secondary vein branching

In globular embryos, *RPK2* is expressed in the outermost layer that gives rise to the ground tissues, an expression pattern that is maintained during the following developmental stages of embryogenesis (Fig. 5) (Nodine et al., 2007). Previous studies have suggested that *RPK2* is required to exclude vascular identity from the protodermis of globular embryos (Nodine et al., 2007). Here, we provide evidence that RPK2 activity is not only necessary to exclude vascular identity in the future epidermis, but also necessary to modulate the branching of secondary veins (Fig. 5). The activity of RPK2 in controlling the development of cortical and endodermal cells has been explained mostly by the perception of CLE17 (Racolta et al., 2018). Within the root stele, CLE45 sensing by RPK2 confers developmental plasticity to companion and protophloem cells in order to safeguard phloem functionality by re-establishing a correct phloem pattern in case the original one fails to form (Gujas et al., 2020). However, our analysis implies that the suppression of cotyledon proximal branching by RPK2 is independent of vascular-specific CLE peptides (Fig. S5), suggesting a differential mechanism between vascular formation in the shoot and the root. Our results indicate that the negative RPK2-mediated control of vein patterning is independent of auxin. Yet, plant cells need to integrate PAT cues and RPK2 signaling to coordinate the establishment and maintenance of vascular tissues. Considering that *rpk2* sensitivity to the blockage of PAT by NPA was not perturbed (Fig. S4), additional factors other than RPK2 may act as a hub to integrate polar auxin transport cues with RPK2-mediated signaling. *RPK2* expression ends at the cell file, delineating the border between the cotyledon margin and the vein cells, in a region where the positive vascular regulator OPS is localized before the establishment of a closed vein network (Fig. 5L,L′, Fig. 7I-K). Although future studies will be required to elucidate whether the mutually exclusive presence of OPS and RPK2 is required to trigger proximal vein branching, it is possible that OPS contributes to the integration of PAT cues with RPK2 signaling through its interaction with VASCULATURE COMPLEXITY AND CONNECTIVITY (VCC) (Roschzttardtz et al., 2014). VCC contributes to the spatiotemporal modulation of PAT during cotyledon vein formation by delivering PIN1 to the vacuole for protein degradation (Yanagisawa et al., 2021). Alternatively, RPK2 may modulate the perception or response of other non-cell-autonomous-acting hormones such as brassinosteroids (BRs), the receptors of which have been involved in the regulation of leaf venation. In particular, BRI1-LIKE2 (BRL2) appears to modulate cotyledon vein patterns through its interaction with the vascular-specific adaptor proteins VIT and VIK (Ceserani et al., 2009). Although RPK2 extracellular domains have been reported to bind only BRASSINOSTEROID INSENSITIVE 1 (Smakowska-Luzan et al., 2018), further experiments may reveal whether RPK2 acts as a hub excluding BR cues from the cotyledon margin. Taken together, our results reveal a molecular genetic framework through which plants at the late stages of embryogenesis modulate the tissue complexity of their vascular networks. Although the function of *RPK2* is conserved in cylindrical and foliar organs, its regulation appears to be tissue specific, comprising unique molecular mechanisms to optimize the functionality of vascular tissues to the constantly changing shape of the organs to which they belong.

## MATERIALS AND METHODS

### Plant material and growth conditions

*Arabidopsis* ecotype Columbia-0 was used as a WT control in all cases, except for experiments related to *pin1-3*, *pin1-5* and and *clv3-7*, in which Landsberg erecta (Ler) was used as a control. Seeds of *pin1-3*, *pin1-5*, *pin1*,*3*,*6*,*4*,*7*,*8* and *PIN1::PIN1-GFP* were kindly provided by Dr Jiri Friml (Institute of Science and Technology, Austria), Dr Miguel A. Perez-Amador (Institute for Plant Molecular and Cellular Biology, Valencia, Spain) and Dr Enrico Scarpella (University of Alberta, Canada), and seeds of *aux1-21 lax1* were provided by Dr Christian Fankhauser (University of Lausanne, Switzerland). *cle45.cr2* and *clv3* loss-of-function mutants were provided by Dr Takashi Ishida (Kumamoto Health Science University, Japan) and Dr Olivier Hamant (ENS Lyon, France), respectively. The transgenic lines *SHR::SHR-GFP* and *MP::MP-GFP* were provided by Dr Joop Vermeer (University of Neuchâtel, Switzerland) and Dr Dolf Weijers (Wageningen University, the Netherlands), respectively. The *CVP2::NLS-3×VENUS*, *CVL1::NLS-3×VENUS*, *CVP2::GUS*, *CVL1::GUS*, *cvp2* and *cvp2 ops* lines have been previously described (Rodriguez-Villalon et al., 2014, 2015; Gujas et al., 2020; Carland and Nelson, 2009). Likewise, the *rpk2*, *cvp2 cvl1*, *amiRPK2 cvp2 cvl1* and *ops* lines have been reported elsewhere (Gujas et al., 2020). The *MP::MP-GFP*, *SHR::SHR-GFP*, *DR5::NLS-VENUS*, *PIN1::PIN1-GFP*, *AUX1::AUX1-YFP* and *OPS::OPS-GFP* translational fusion reporter lines in distinct genotypes were obtained by crossing these lines with the indicated loss-of-function mutants or have been previously published (Rodriguez-Villalon et al., 2015). Seeds were surface sterilized, stratified at 4˚C and grown vertically on half-strength (0.5×) Murashige and Skoog (MS) medium-containing plates under standard continuous-light growth conditions. Seedlings were transferred to soil and grown in 5 cm×5 cm pots in peat-based compost medium in a walk-in chamber maintained at a constant temperature of 23°C and 65% humidity for a 16 h photoperiod and with a light intensity of 250 μmol photons m^−2^ s^−1^ until flowering, when siliques were collected to extract embryos.

### Cloning and plant transformation

All constructs were generated using double or triple Multi-Site Gateway system (Thermo Fisher Scientific) following the manufacturer's instructions. To clone the *ABI3* promoter, a 2 kb genomic DNA region was PCR amplified and introduced via pDNRP4-P1r (Invitrogen) to generate pENTRY-ABI3. *CLV3* and *CLE45* were amplified using the following primers: CLV3_XmaI_F, 5′-TAACCCGGGATGGATTCGAAGAGTTTTCTGC-3′; CLV3_SpeI_R, 5′-CGCCACTAGTTCAAGGGAGCTGAAAGTTGT-3′; CLE45_XmaI, 5′-TAACCCGGGATGTTGGGTTCCAGTACAAGA-3′; CLE45_SpeI_R, 5′-CGCCACTAGTTTAAGAAAATGGCTGAGCTTTGT-3′. The PCR products were cloned into pENTRY vectors using standard procedures and further recombined together with pENTRY-ABI3 into the destination vector pEDO 097 (provided by Dr Joop Vermeer). The *BAM3* promoter (2139 bp) was amplified using the following primers: pBAM3_attB4_F, 5′-GGGGACAACTTTGTATAGAAAAGTTGCCCTGCTTCCCTAGTTTATCTAATAAATCTGATG-3′, and pBAM3_attB1r_R, 5′-GGGGACTGCTTTTTTGTACAAACTTGGTGTAACATCAGAAAAATAAAAACAAAAATTTGTCC-3′. It was then fused with the *NLS-3×VENUS* construct as previously described (Gujas et al., 2020). For *PIN1* silencing, an artificial microRNA (amiRNA) was designed *in silico* by using the software P-SAMS (Fahlgren et al., 2016). To obtain the construct, the following primers were used: CAM046, 5′-TGTATCCTTGCGGCAAAGCTGCCTCATGATGATCACATTCGTTATCTATTTTTTGAGGCAGCTTGGCCGCAAGGA-3′, and CAM047, 5′-AATGTCCTTGCGGCCAAGCTGCCTCAAAAAATAGATAACGAATGTGATCATCATGAGGCAGCTTTGCCGCAAGGA-3′. The miRNA was then introduced into the pMDC32B-AtMIR390a-B/c plasmid (Addgene plasmid #51776) by performing a digestion-ligation reaction, as previously described (Carbonell et al., 2014). Transgenic plants were generated using floral-dip transformation techniques as previously described (Gujas et al., 2017).

### Confocal microscopy

Mature embryos were dissected from seed coats and stained using the mPS-PI method according to Truernit et al. (2008), and then imaged using a ZEISS LSM 780 confocal microscope. Embryos in globular stage and onwards were dissected from siliques and fixed in 75% ethanol and 25% acetic anhydride for 24 h at 4°C. After the fixing step, embryos were washed, incubated in 1% periodic acid (Sigma-Aldrich, 10450-60-9) and rinsed with water following incubation in mPS-PI. Finally, embryos were mounted on glass slides in chloral hydrate (Sigma-Aldrich, 302-17-0). Embryos with fluorescent reporters were imaged using Renaissance staining SR2200 (Renaissance Chemicals) as previously described (Smit et al., 2020). Pictures showing the localization of protein in the roots were obtained using a two-photon laser Leica SP8 microscope. Six-day-old roots were stained for 5 min in a 10 µg/ml aqueous solution of propidium iodide. For esthetic reasons, images were rotated and displayed with a matching background. All image processing was performed using ImageJ software. Procambial cell file width was measured for individual procambial cells belonging to the midvein using ImageJ and represented as the mean before and after distal branching. Midvein width was measured as the total width of all procambial cell files comprising the midvein before and after distal branching. Intensity ratios to assess polar protein distribution in procambial and protophloem cells were quantified by measuring the mean intensity for each region of interest (ROI) using ImageJ. Basal, apical and lateral membrane signals were normalized against the total ROI area. The mean of all cells quantified is represented as the intensity ratio of each protein in the corresponding genotype.

### mRNA sequencing of *Arabidopsis* embryos and data analysis

Fifty torpedo stage embryos from each genetic background, WT, *cvp2 cvl1* and *pRPK2::amiRPK2 cvp2 cvl1*, were manually dissected out of the ovule with needles and immediately placed in TRIzol reagent (Ambion) and ground with a sterile pestle. Two or three biological replicates (50 embryos) were collected and analyzed. All samples were frozen in TRIzol and kept at −80°C until RNA extraction. RNA extraction was completed by incubating samples at 60°C for 30 min and then purified according to Palovaara et al. (2017). The resulting RNA was cleaned up and concentrated using the RNeasy MinElute Cleanup Kit (Qiagen, 74204), as described Palovaara et al. (2017). Samples were eluted with 14 μl RNase-free water and stored at −80°C. Isolated RNA displayed an RNA integrity number ranging from 6.8 to 7.7. mRNA-sequencing libraries were generated with the Smart-Seq2 kit (Agilent) and subsequently sequenced in an Illumina NovaSeq 6000 at the Functional Genomics Center Zurich. Data have been stored on the Gene Expression Omnibus (GEO) with accession number GSE178241. Quality validation was carried on using FastQC (https://www.bioinformatics.babraham.ac.uk/projects/fastqc/), after which adapter sequences were removed using Trimmomatic (v0.39, Bolger et al., 2014) with the options SE -phred33 ILLUMINACLIP:trimmed/NexteraPE-PE.fa:2:30:10 SLIDINGWINDOW:4:15 MINLEN:50. Trimmed reads were then aligned onto *Arabidopsis thaliana* TAIR10 Ensembl genome and genes annotation (retrieved from igenome) using HISAT2 (v2.2.1, Kim et al., 2019) with options -k 10 --max-intronlen 1000--known-splicesite-infileTAIR10_splicesites.txt after applying hisat2_extract_splice_sites to TAIR10 Ensembl genes annotation. Reads count table for annotated genes was generated using the featureCounts function (v2.0.1) from the Subread package (Liao et al., 2014) with options -O -M -T 10—largest Overlap--minOverlap 10 --primary. Differential analysis was performed using DEseq2 (v1.22.2, Love et al., 2014). Genes with an adjusted *P*-value (*P*_adj_) lower than 0.05 were considered as differentially expressed.

### Reverse transcription quantitative PCR (RT-qPCR)

Total RNA was isolated with a RNeasy Plant Mini Kit (Qiagen) according to the manufacturer's instructions. cDNA was prepared from 2 µg of total RNA with the RevertAid First Strand cDNA Synthesis Kit (Thermo Fisher Scientific) according to the manufacturer's instructions. The resulting cDNA was diluted 1:10 in ddH_2_O and 2 µl of the resulting dilution was used in the PCR reaction. The qPCR reaction solution was prepared using KAPA SYBR FAST qPCR mix (Thermo Fisher Scientific). All reactions were performed in triplicate and expression levels were normalized to those of *PDF2*. The following primers were used: *PDF2*_Fw, 5′-TAACGTGGCCAAAATGATGC-3′; *PDF2*_Rv, 5′-GTTCTCCACAACCGCTTGGT-3′ (Czechowski et al., 2005); *AUX1*_Fw, 5′-GGATGGGCTAGTGTAAC-3′; *AUX1*_Rv, 5′-TGACTCGATCTCTCAAAG-3′ (Dindas et al., 2018); *PIN1*_Fw, 5′-ACAAAACGACGCAGGCTAAG-3′; *PIN1*_Rv, 5′-AGCTGGCATTTCAATGTTCC-3′ (Heisler et al., 2005); *OPS*_Fw, 5′-GACAGGTCTAGTAGCTCCATGAGG-3′; *OPS*_Rv, 5′-AGCTTTGGCTCGTCCATATCCG-3′. *OPS* primers were designed using QuantPrime.

### Histology and β-glucuronidase (GUS) staining

Developing cotyledons were imaged at 7 or 8 days as indicated. Cotyledons and leaves were fixed with 3:1 ethanol:acetic acid, and their clearing was performed by dehydration in 80% ethanol, followed by treatment with 10% sodium hydroxide for 1 h at 37°C. Cotyledons and leaves were then mounted in 50% glycerol. Black and white images were captured and the brightness and contrast adjusted using ImageJ. To visualize GUS staining, we used a staining buffer containing 2 mM 5-bromo-4-chloro-3-indolyl-β-D-glucuronide and 1 mM potassium ferricyanide and ferrocyanide as previously described (Carland and Nelson, 2004). We considered BPs as lateral/secondary veins bifurcated from both midvein and distal secondary veins (see model shown in Fig. 1O).

### Seedling treatments

To perform NPA and CLE treatments, 4-day-old seedlings grown in MS medium were transferred to a medium supplemented with or without NPA (10 μM), CLV3 (5 nM), CLE45 (20 nM), CLE25 (100 nM) and CLE26 (150 nM) for 5 days. The quantification of the root sensitivity to NPA treatment was performed by measuring the length of the primary root using ImageJ.

### Statistical analyses

Microsoft Excel and R were used for statistical analyses. Statistical significance was calculated using two-tailed paired Student’s *t*-tests. Differential analysis of the RNA-sequencing data was performed using DEseq2 (v1.22.2) and *P*-values for the Venn diagrams and MA plots were corrected (*P*_adj_) for multiple testing using the Benjamini and Hochberg method. For heatmaps, the *P*-values were used as indicated in the figure legend.

## Supplementary Material

Supplementary information

Reviewer comments
