# Supplementary material for: A genetic framework for proximal secondary vein branching in the Arabidopsis thaliana embryo
Source: Development. 2022 Jun 27;149(12):dev200403. doi: 10.1242/dev.200403 (PMC9270971; doi:10.1242/dev.200403)
Supplement: Supplementary information [file develop-149-200403-s1.pdf]

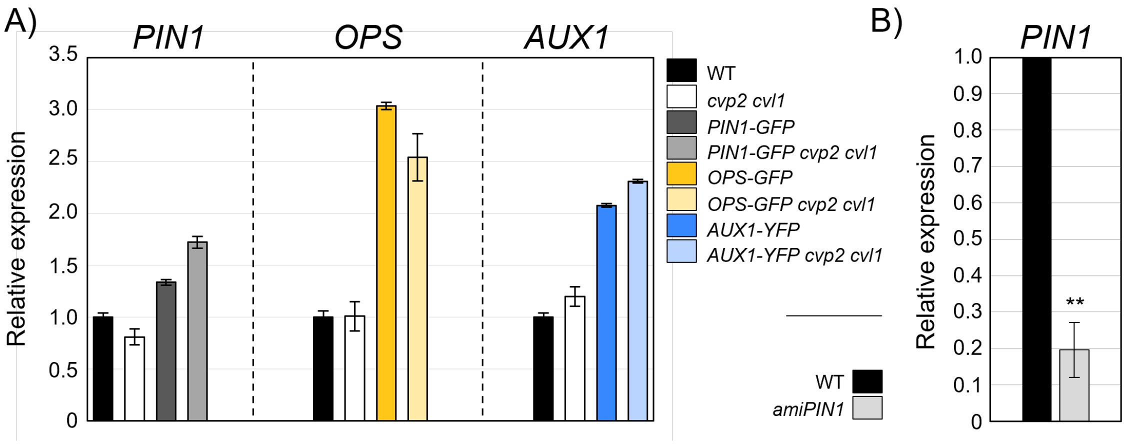

Fig. S1. Transcriptional profiling of the transgenic lines used in the current study. A) Levels of *PIN1*, *OPS* and *AUX1* normalized expression in 7-day-old seedlings of the indicated genotypes in comparison to wild type. Values represent the mean of 3 technical replicates and error bars represent the standard deviation of these replicates. **B)** Levels of *PIN1* in 7-day-old seedlings of WT (Col-0) and *amiPIN1*. Values represent the mean of 2 biological replicates and error bars represent the standard deviation of these replicates. ** indicates $p<0.01$ (Student's t-test).

Development • Supplementary information

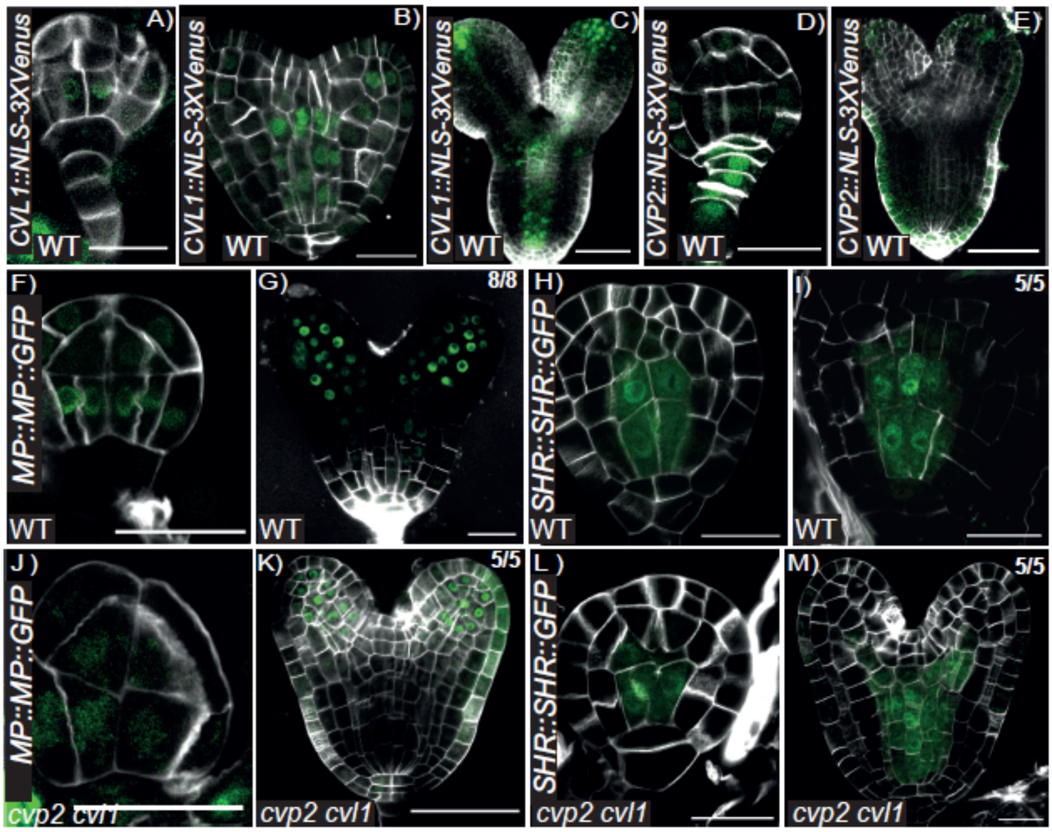

Fig. S2. Analysis of *CVP2* and *CVL1* expression and vascular identity domains in *cvp2 cvl1* embryos. A-E) Expression pattern of the indicated genes in early globular (A, D), heart (B, E) and torpedo (C) developmental stages. Renaissance SR2200 staining highlights patterning divisions by labelling plant cell walls. Scale bars represent 50µm in A, C, D, E and 20µm in B. **(F-I)** Expression pattern of the indicated vascular genes in globular (F, H, J, L) and heart stage (G, I, K, M) embryos of WT and *cvp2 cvl1*. Scale bars represent 20µm in (G, I, K, M) and 50µm in (F, H, J, L).

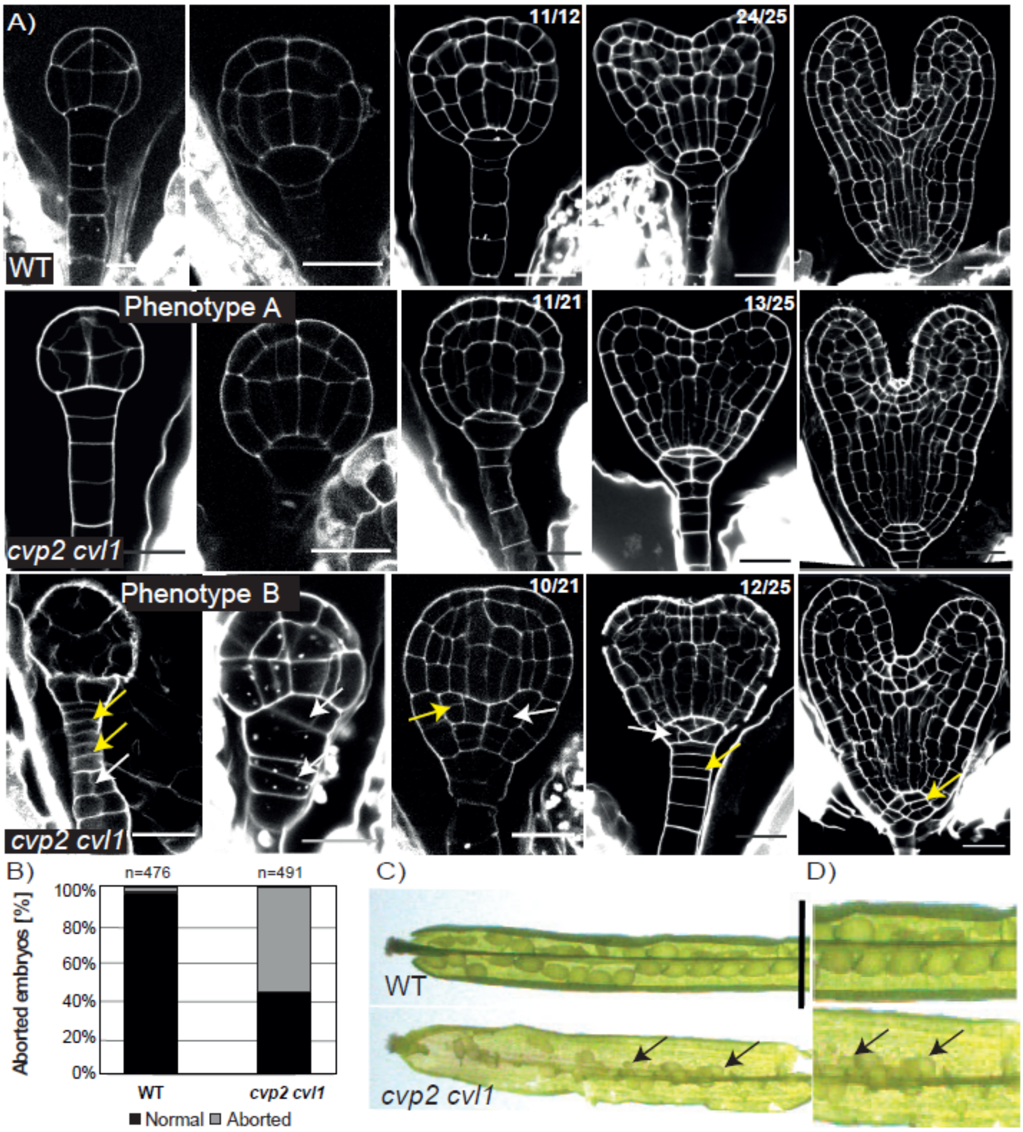

Fig. S3. *cvp2 cvl1* embryos exhibit aberrant divisions. A) Embryo morphology of WT compared to *cvp2 cvl1* embryos using mPS-PI on ovules taken from green siliques and imaged using confocal microscopy. *cvp2 cvl1* shows aberrant divisions at all stages of embryogenesis but most abundantly during the globular stage. Yellow arrows point to extra divisions, whereas white arrows show divisions in the wrong orientation. Scale bars are 20µm or 50µm. **B)** Graphical representation of the percent of aborted embryos occurring in *cvp2 cvl1* vs. WT siliques numbered 2 and 3 counted from the apical meristem (containing globular stage embryos). **C)** Image of a dissected WT silique as compared to *cvp2 cvl1* under bright field using a stereomicroscope. Scale bar represents 1inch.

Development • Supplementary information

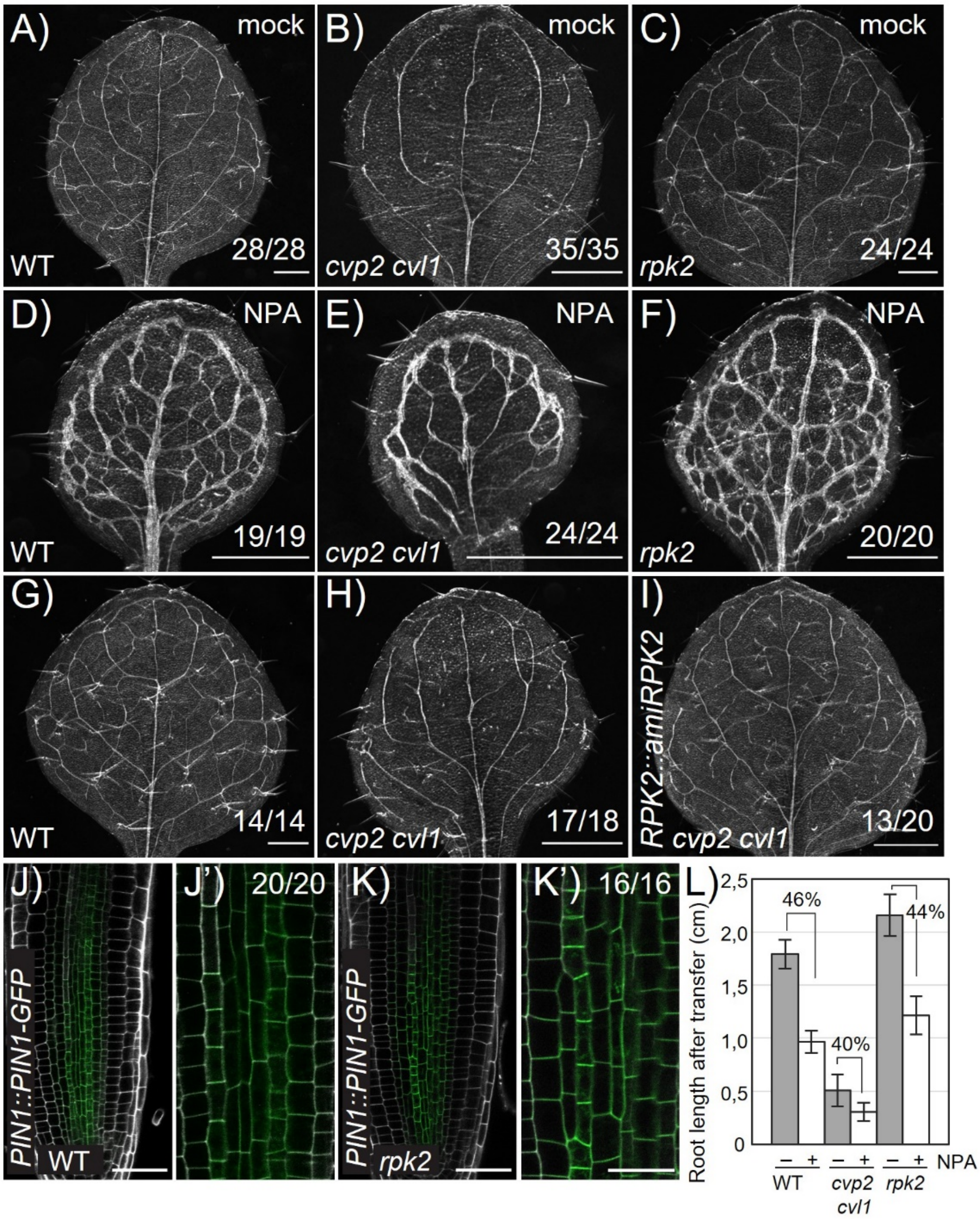

Fig. S4. Sensitivity to PIN1-mediated auxin transport is not disturbed in *rpk2*. A-F) Analysis of cleared leaves of seedlings grown for 4 days (until clear emergence of cotyledons could be detected) transferred to a media supplemented with 10 µM NPA or mock conditions for 5 days. n = 19-35 for each genotype. Scale bars: 500 µm. **G-I)** Cleared leaves of the indicated genotypes imaged with a stereomicroscope in bright field on a black background. n= 14-20 for each genotype. **J-K')** Confocal microscopy analysis of *PIN1::PIN1-GFP* distribution in cells of the root stele of WT and *rpk2-2* seedlings. J' and K' represent magnification of the region shown in J and K. Scale bars represent 50µm in J,K and 20µm in J' and K'. **L)** Root length of seedlings grown as described in A-F) were measured. Note that root length was measured after the treatment with NPA or mock. The root length of seedlings treated with mock were set to 100% and the % shown in the graph represents the % of inhibition of root length by NPA. n=39-56.

Development • Supplementary information

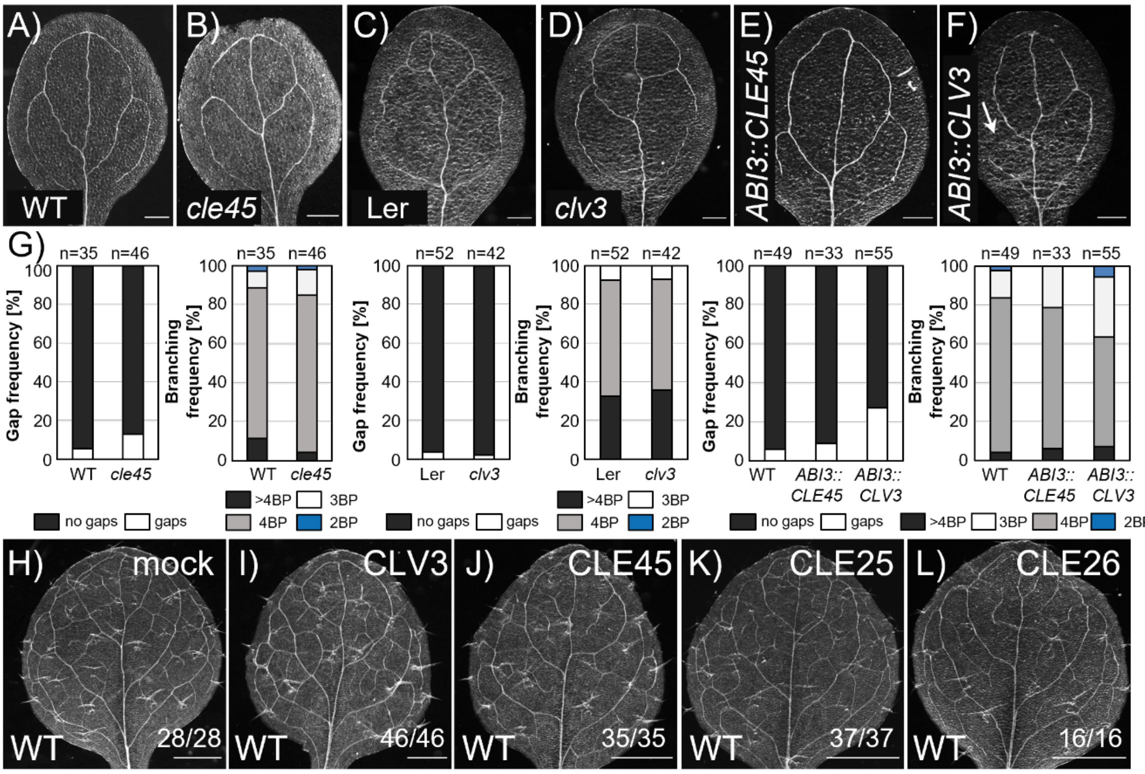

Fig. S5. RPK2 modulation of vascular branching is independent of vascular-specific CLE peptides. A-F) Analysis of the continuity and complexity of cotyledon vein network in 8-day-old seedlings of the indicated genetic backgrounds. Arrow in F indicates defects in the tip-to-base proximal branching in *pABI3::CVL3*. Scale bars represent 200µm. **G)** Quantification of the gap and branching frequency in the cotyledons analyzed in A-F. n= 33-55 for each genotype. **H-L)** Analysis of the vein pattern in cleared leaves of 9-day-old seedlings transferred to a medium supplemented with the indicated CLE peptides once the emergence of the cotyledons could be detected. n= 16-46 for each genotype. Scale bars represent 500µm.

Development • Supplementary information

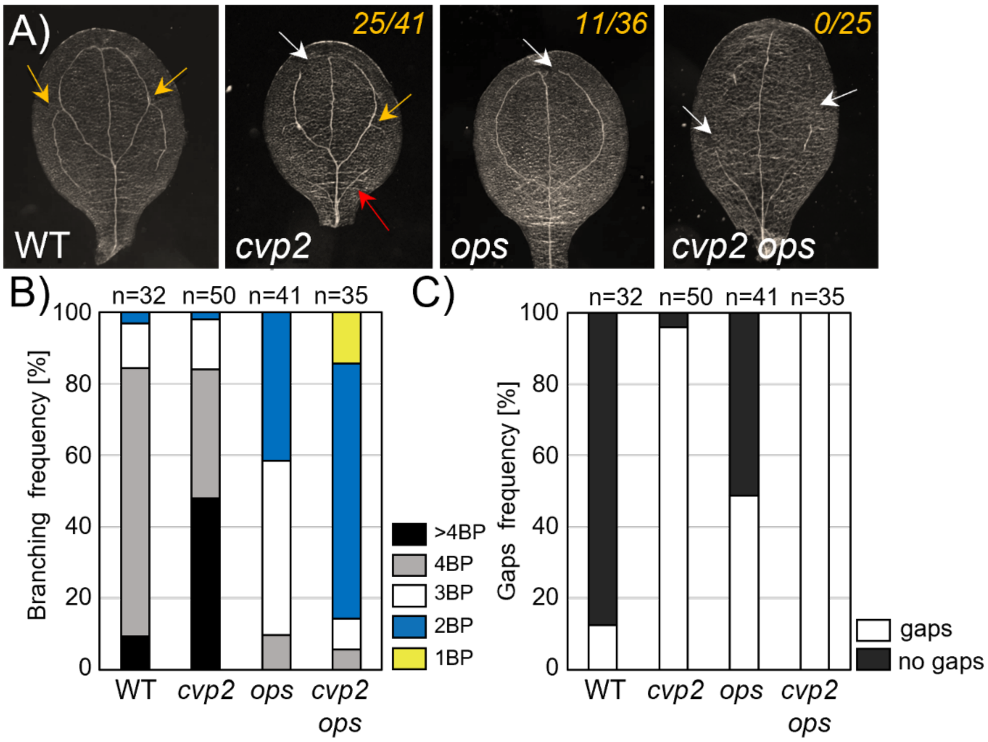

Fig. S6. Genetic interaction between OPS and CVP2 in cotyledon vein patterning. A) Representative images of 7-day-old cleared cotyledons of the indicated genotypes. Yellow arrows mark secondary proximal branching points formed in a tip-to-base manner, while red arrows indicate secondary proximal branching points formed in the opposite direction. White arrows mark gaps or vascular discontinuities. The numbers indicate the frequency of the appearance of proximal secondary veins in a tip-to-base manner. Note that these numbers include only those cotyledons where the loops are not complete and then is possible to discriminate between proximal secondary veins originated in a tip-to-base or in a base-to-tip manner. **B-C)** Quantification of branching (B) and gaps (vascular discontinuities) frequency (C) in the indicated genotypes. n=32-50 for each genotype. BP: branching points, counted as the initiation (even if not completed) of a new secondary vein.

Development • Supplementary information

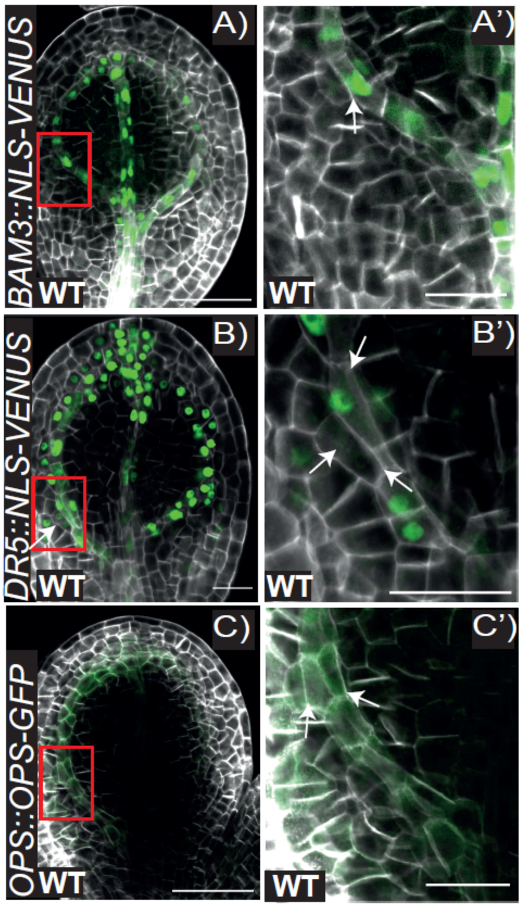

Fig. S7. A periclinal division occurs at the branching point. Representative pictures of early torpedo stage embryos harbouring *BAM3::NLS-3xVENUS* (A), *DR5::NLS-VENUS* (B) *and OPS::OPS-GFP* (C). Embryonic cotyledons were stained with Renaissance stain SR2200 and visualized by confocal microscopy. Magnification of the branching region squared in A), B) and C) is shown in A'), B') and C'), respectively. White arrows indicate nuclei in the cells having undergone a periclinal cell division at the branching point. Scale bars represent 50 µm in (A, C) and 20 µm in (A', B, B', C').
