# Supplementary material for: A genetic framework for proximal secondary vein branching in the Arabidopsis thaliana embryo
Source: Development. 2022 Jun 27;149(12):dev200403. doi: 10.1242/dev.200403 (PMC9270971; doi:10.1242/dev.200403)
Supplement: Reviewer comments [file develop-149-200403_review_history.pdf]

A genetic framework for proximal secondary vein branching in the *Arabidopsis thaliana* embryo

Elizabeth Kastanaki, Noel Blanco-Touriñán, Alexis Sarazin, Alessandra Sturchler, Bojan Gujas, Francisco Vera-Sirera, Javier Agustí and Antia Rodriguez-Villalon
DOI: 10.1242/dev.200403

Editor: Ykä Helariutta

Review timeline

Original submission:	30 November 2021
Editorial decision:	14 January 2022
First revision received:	8 April 2022
Accepted:	9 May 2022

Original submission

First decision letter

MS ID#: DEVELOP/2021/200403

MS TITLE: A genetic framework for proximal secondary vein branching in the *Arabidopsis thaliana* embryo.

AUTHORS: Elizabeth Kastanaki, Noel Blanco-Touriñán, Alexis Sarazin, Alessandra Sturchler, Bojan Gujas, Francisco Vera-Sirera, Javier Agustí and Antia Rodriguez-Villalon

I have now received all the referees' reports on the above manuscript, and have reached a decision. The referees' comments are appended below, or you can access them online: please go to BenchPress and click on the 'Manuscripts with Decisions' queue in the Author Area.

As you will see, the referees express considerable interest in your work, but have some significant criticisms and recommend a substantial revision of your manuscript before we can consider publication. If you are able to revise the manuscript along the lines suggested, which may involve further experiments, I will be happy receive a revised version of the manuscript. Your revised paper will be re-reviewed by one or more of the original referees, and acceptance of your manuscript will depend on your addressing satisfactorily the reviewers' major concerns. Please also note that Development will normally permit only one round of major revision.

We are aware that you may be experiencing disruption to the normal running of your lab that make experimental revisions challenging. If it would be helpful, we encourage you to contact us to discuss your revision in greater detail. Please send us a point-by-point response indicating where you are able to address concerns raised (either experimentally or by changes to the text) and where you will not be able to do so within the normal timeframe of a revision. We will then provide further guidance. Please also note that we are happy to extend revision timeframes as necessary.

Please attend to all of the reviewers' comments and ensure that you clearly highlight all changes made in the revised manuscript. Please avoid using 'Tracked changes' in Word files as these are lost in PDF conversion. I should be grateful if you would also provide a point-by-point response detailing how you have dealt with the points raised by the reviewers in the 'Response to Reviewers' box. If you do not agree with any of their criticisms or suggestions please explain clearly why this is so.

© 2022. Published by The Company of Biologists under the terms of the Creative Commons Attribution License (https://creativecommons.org/licenses/by/4.0/).

Reviewer 1

Advance summary and potential significance to field

In this study, performed by Kastanaki and colleagues, the authors investigate the process of vein formation in leaf-like organs of Arabidopsis thaliana. Starting from the well established discontinuity of the vein network in the cvp2 cvl1 double mutant, the authors perform a careful analysis of vein formation during embryogenesis focusing on distal and proximal loop formation. Comparing wild type with cvp2 cvl1 double mutants shows that discontinuity is not observed yet at very early stages but presumably emerges during strand extension from heart to torpedo stage. The authors also propose that distal veins diverge directly from the midvein whereas proximal veins emerge from periclinal cell divisions initiating branching points. Based on their phenotypic analyses the authors conclude that CVP2 and CVL1 are especially involved in the latter process and that their role is largely independent from regulating auxin transport. Instead, as previously shown for the root tip, both genes genetically interact mainly with RPK2 repressing the formation of vascular strands also in the context of cotyledons and weakening the cvp2 cvl1 (and ops) phenotype and transcriptome aberrations when mutated.
I rate this study as being very well conducted, the amount of accumulated data is immense and I do have hardly any concern with regard to the experimental strategy or data generation/presentation. The question toward the formation of plastic vein patterning in plant leaves, is certainly fundamental and served as a paradigm for pattern formation in the past. Moreover, performing these analyses during embryogenesis is technically challenging and has (as far as I am aware) not been performed before. Therefore, the study allows insights into the process with an unprecedented depth and amount of information. In this regard, the authors make efficient use of their analytical skills and of existing technical and genetic resources.

Comments for the author

I have, however, some reservation about several conclusions the authors make. In this regard, I was not always able to follow their line of argumentation and sometimes conclusions and presented data seem to be disconnected. It could be that this is due to the fact that the authors anticipate too many connections on the side of the reader and, with better explanation, this issue can be solved. I also think a cvp2;cvl1;ops triple mutant would be important to analyse if the authors want to keep this part in this paper. Alternatively, the interaction between CVP2, CVL1 and OPS can be removed as it does not add much to the main message of the paper and, as I said, the manuscript is very data-rich already. In detail, my points are the following.
1) Line 112: ‘distal’ -> ‘proximal’?
2) Line 114: With regard to the terms ‘before’ and ‘after’, it is unclear whether they are used in a temporal or spatial sense. I believe, that that the authors use them in a spatial sense, but this was difficult to figure out. I recommend revising the wording in this case. The same is when the authors use the term ‘diverge’. Does diverge mean that veins split during leaf growth forming two veins or does this mean that there are branching points at a given time point?
3) Lines 116-118: This is an example where I cannot follow how the authors get to this conclusion. How can the authors conclude that ‘the branching of proximal veins appears to be different and follow a yet-to-be described mechanism’ from what they describe in the text before?
4) Fig.2: Not all arrows are described in the legend.
5) Line 166: It is unclear to me where I see this in the figure.
6) Line 167: Again, I am not sure where I see that in the figure 2S and how something about secondary vein formation can be concluded by looking at early embryogenesis.
7) Line 211-214: Another example. It is hard to connect the conclusions to the reported observations. Also, saying ‘auxin may be necessary to establish continuous veins, but polar transport of auxin does not regulate vein continuity’ seems contradictive and hard to grasp. Here, the authors could maybe come up with clearer statements.
8) Fig. 5D: Although quantifications (Fig. 5E) suggests zero vein gaps in cvp2;cvl1;amiRPK2 plants, in Fig.5D there are clearly gaps visible. How does this fit together?
9) Line 234: ‘greatest’ -> ‘strongest’
10) Line 235-236: It is unclear to me on what ground this statement is made.
11) Line 253: ‘excluding’ -> ‘arguing against’ I don’t think an altered genetic program of mesophyll cells is completely excluded here.

© 2022. Published by The Company of Biologists under the terms of the Creative Commons Attribution License (https://creativecommons.org/licenses/by/4.0/).

12) Line 340-341: I think to be able to make this statement, a cvp2;cvl1;ops triple mutant is required. Here, I would expect an enhancement of the gap phenotype.

Reviewer 2

Advance summary and potential significance to field

In the manuscript by Kastanaki and co-workers, the authors describe a logical set of experiments to understand the molecular players involved in controlling secondary vein formation in Arabidopsis embryonic leaves. Despite the importance of vascular development, most efforts are focussed on root tissues; likely due to the more predictable organisation and division patterns. This makes the insights described in this manuscript important as they add to the very limited molecular knowledge about this process during leaf development. In this aspect, I believe this manuscript can be of interest to a larger group of scientists.
Although I agree with the general conclusions of this manuscript which is well written; there are some aspects where the author's input seems required. These are listed below in chronological order.

Comments for the author

Line18: what are plate meristematic cells? Can this be defined in the text for the non-expert readers?
Line92: perhaps a semantics discussion, but I thought that procambium cells in the early embryo are called pre-procambium?
Line96: is there a quantification to identify these elongated narrow cells (cells are 2x as long and narrow, for example, comparing to mesophyll cells), or this is rather determined visually?
Line116 and Fig 1S-U: number of samples is not indicated in the figure nor the legend. Related, it would be better to use box plots with indication of the datapoints instead of bar charts which give limited information.
This should be done throughout the manuscript. Panel U does not even have an Y- axis or error bars. The authors could also consider to homogenise the graph layouts throughout as the three in Figure 1 already are all different in size, layout etc. Related to this and more toward small details, the arrangement of the figure panels is messy with different spaces between the panels etc. Although not at all relevant to the science in this manuscript, it does give a bit of a messy appearance and does not match with the overall high quality of imaging presented in this work.
Line125: The intact midvein in the double mutant is not very clearly observed from Fig2B. Perhaps a higher magnification and quantification would help?
Fig2E: the indication of 1/30 is confusing as it would suggest only 1/30 looks like WT. I assume the authors mean that 29/30 look normal in WT?
Line151: please quantify the phenotype A and B in Fig S3. In panel B, what is n? Please indicate numbers of samples throughout all figure panels.
Line 175: it does appear mostly basal, however I would not call PIN1 localisation in the mutant completely normal, as it seems that there is a higher amount of intracellular PIN1 aggregation. Can the authors explain what this might mean? Perhaps it would also be interesting to compare plasma membrane/intracellular signal intensity.
Fig3: no statistics in the graphs, no indication of number of samples etc. Please indicate this information throughout. Panels M, N: labels are not clear for the last two bars. Are we seeing PIN1-PIN1-GFP?
Line 188: both pin1-3 and pin1-5 are in the Ler background. It is interesting that Ler background lines behave so differently concerning their branching frequency in comparison to Col-0 lines. Why would this be?
Line196: increasing PIN1 dosage to see effect would be clearer with a 35S/overexpression promoter fusion.
Line 199: what phenotype would a higher order mutant (lacking functional PIN1, CVP2, CVP3) exhibit? A cross with the auxin mutants would shed light on the role in distal branching formation in the cvp2 cvl1 double mutant
Line229: quantify 'occasionally'
Line236: RPK2 involvement would be clear if ectopic expression would be observed in the cvp2cvl1 double mutant at the branching points. The authors should provide additional explanations or

© 2022. Published by The Company of Biologists under the terms of the Creative Commons Attribution License (https://creativecommons.org/licenses/by/4.0/).

hypotheses on how RPK2 can influence branch points non-cell autonomously, specifically if auxin is not involved as shown in the next paragraph.
Line 285: Obviously the authors also realise that a polar distribution in the stele does not suggest automatically that it is also polar in the leaf veins? I feel that to say that this process is PIN1 independent one would have to show PIN1:GFP or DR5 or something in the RPK2 mutants or simply show polarity in the correct place for which conclusions are drawn. This should be possible, even with low fertile plants. Related and out of curiosity, what would be the expression pattern of RPK2 in the pin1 mutant?
Line333: could RPK2 and OPS interaction perhaps at the sites of overlapping expression? What would the implication be?
Line 336: does the ops mutant show a defect in branching?
Line 341: Do higher order mutant combinations between OPS, RPK2, CVP2 or CVL1 exist? If so, it would be good to see whether they are in the same pathway/epistatic/additive/etc. I can however imagine that it would be difficult to get these higher order mutants due to lethality.
Line399: according to Sup Fig 1, introduction of PIN1:PIN1-GFP hardly increased (not even two-fold, in fact its comparable to control PIN1-GFP line) dosage in the mutant. Perhaps using a different promoter would be more useful, otherwise it can be hard to claim that it does not enhance branching based on what is shown here.

First revision

Author response to reviewers' comments

RESPONSE TO REVIEWERS

We are very grateful about your comments and suggestions, which we believe have helped us to detect the weak points of our manuscript and improved it. Please find in blue font the answers to each of your requests and suggestions. Any modification of the main text appears in red font (in here and in the manuscript).

Reviewers' comments:

Reviewer #1

In this study, performed by Kastanaki and colleagues, the authors investigate the process of vein formation in leaf- like organs of Arabidopsis thaliana. Starting from the well established discontinuity of the vein network in the *cvp2 cvl1* double mutant, the authors perform a careful analysis of vein formation during embryogenesis focusing on distal and proximal loop formation. Comparing wild type with *cvp2 cvl1* double mutants shows that discontinuity is not observed yet at very early stages but presumably emerges during strand extension from heart to torpedo stage. The authors also propose that distal veins diverge directly from the midvein whereas proximal veins emerge from periclinal cell divisions initiating branching points. Based on their phenotypic analyses the authors conclude that CVP2 and CVL1 are especially involved in the latter process and that their role is largely independent from regulating auxin transport. Instead, as previously shown for the root tip, both genes genetically interact mainly with RPK2 repressing the formation of vascular strands also in the context of cotyledons and weakening the *cvp2 cvl1 (and ops)* phenotype and transcriptome aberrations when mutated. I rate this study as being very well conducted, the amount of accumulated data is immense and I do have hardly any concern with regard to the experimental strategy or data generation/presentation. The question toward the formation of plastic vein patterning in plant leaves, is certainly fundamental and served as a paradigm for pattern formation in the past. Moreover, performing these analyses during embryogenesis is technically challenging and has (as far as I am aware) not been performed before. Therefore, the study allows insights into the process with an unprecedented depth and amount of information. In this regard, the authors make efficient use of their analytical skills and of existing technical and genetic resources.

© 2022. Published by The Company of Biologists under the terms of the Creative Commons Attribution License (https://creativecommons.org/licenses/by/4.0/).

I have, however, some reservation about several conclusions the authors make. In this regard, I was not always able to follow their line of argumentation and sometimes conclusions and presented data seem to be disconnected. It could be that this is due to the fact that the authors anticipate too many connections on the side of the reader and, with better explanation, this issue can be solved. I also think a *cvp2;cvl1;ops* triple mutant would be important to analyse if the authors want to keep this part in this paper. Alternatively, the interaction between CVP2, CVL1 and OPS can be removed as it does not add much to the main message of the paper and, as I said, the manuscript is very data- rich already. In detail, my points are the following.

We thank this reviewer for bringing this to our attention. In the current version of the manuscript, we have tried to improve our line of argumentation (for more details in relation to the changes, please see changes in the manuscript in red). We do agree that the analysis of *cvp2 cvl1 ops* would be interesting for the paper. In this triple mutant we would expect to see an equally impaired or even more severe cotyledon vascular network than in *cvp2 cvl1*. However, it wouldn't help us to determine whether *OPS* is epistatic or not to *CVP2* and *CVL1* in this context. Hence, we have decided to take advantage of *cvp2* single mutants (described in Rodriguez-Villalon et al., Development 2015), which shows a milder defective vascular phenotype than *cvp2 cvl1*, and we crossed it with *ops*. While *cvp2* exhibits mild defects in proximal branching, this phenotype is more severe when additionally knocking out the activity of *ops*. Analysis of the *cvp2 ops* double mutant is now displayed in the new Supplemental Figure 6. The description of its phenotype appears in the text as follows:

"Consistent with these findings, the mild proximal branching phenotype observed in *cvp2* single mutants was more pronounced when introgressed in *ops* genetic background (Fig. S6A-C)."

1) Line 112: 'distal' -> 'proximal'?

In this part of the text, we are trying to characterize the distal vein formation. Therefore, the term distal is correct.

2) Line 114: With regard to the terms 'before' and 'after', it is unclear whether they are used in a temporal or spatial sense. I believe, that that the authors use them in a spatial sense, but this was difficult to figure out. I recommend revising the wording in this case. The same is when the authors use the term 'diverge'. Does diverge mean that veins split during leaf growth forming two veins or does this mean that there are branching points at a given time point?

We do agree with reviewer's comment, especially in the need to clarify that these cell files act as branching points at one point. To do so, we have changed the text as follows:

"Here we observed that the number of cell files that constitutes the midvein is higher in the region where the distal veins hasn't yet branched out (Fig. 1P-S)."

"These observations indicate distal secondary veins directly branch out from the cell files comprised in the midvein, which at one point act as branching points and change their spatial progression."

3) Lines 116-118: This is an example where I cannot follow how the authors get to this conclusion. How can the authors conclude that 'the branching of proximal veins appears to be different and follow a yet-to-be described mechanism' from what they describe in the text before?

We have amended this by changing the text as it is described below:

"Instead, the branching of proximal veins appears not to follow the same pattern, as the number of cell files in the regions adjacent to the branching points remains similar".

4) Fig.2: Not all arrows are described in the legend.

We have included the explanation of all the arrows in the legend.

© 2022. Published by The Company of Biologists under the terms of the Creative Commons Attribution License (https://creativecommons.org/licenses/by/4.0/).

5) Line 166: It is unclear to me where I see this in the figure.

In the figure 2Q it could be observed how the branching of the veins in *cvp2 cvl1*, whose progression occurs in a base- to-tip manner, follows a similar pattern as the one described for distal veins. With a white arrow this is indicated in the main text.

"…procambial cell files appeared to be connected to the basal end of the midvein, arising from the cells comprised in the midvein (Fig. 2Q, marked by a white arrow). "

6) Line 167: Again, I am not sure where I see that in the figure 2S and how something about secondary vein formation can be concluded by looking at early embryogenesis.

As we have shown in the figure, during late embryogenesis the branching of proximal veins already takes place. In our studies we have shown that the activities of both enzymes, CVP2 and CVL1 are necessary to modulate this process. So, we believe that these findings go in line with our claim displayed in line 167.

7) Line 211-214: Another example. It is hard to connect the conclusions to the reported observations. Also, saying 'auxin may be necessary to establish continuous veins, but polar transport of auxin does not regulate vein continuity' seems contradictive and hard to grasp. Here, the authors could maybe come up with clearer statements.

To convey better our messages, we have focused on the role of auxin polar transport and how it seems not to be responsible for the vein defects observed in *cvp2 cvl1*, at least the ones related to branching. We have re-phrased the text as:

"Taken together, our observations suggest that while polar auxin transport modulates distal branching, this is not responsible for the vein discontinuities and proximal branching defects observed in *cvp2 cvl1* embryos."

8) Fig. 5D: Although quantifications (Fig. 5E) suggests zero vein gaps in *cvp2;cvl1;amiRPK2* plants, in Fig.5D there are clearly gaps visible. How does this fit together?

amiRPK2 cvp2 cvl1 plants do not exhibit a continuous vein strand. In the plot mentioned by the reviewer, the gap frequency in these cotyledons is 100% (white boxes).

9) Line 234: 'greatest' -> 'strongest'

We have corrected the text accordingly.

10) Line 235-236: It is unclear to me on what ground this statement is made.

RPK2 appears expressed in the embryonic cotyledons'margins, where cotyledons veins are excluded. Since RPK2 suppress the branching of proximal veins, and its expression is enhanced in *cvp2 cvl1* and expanded towards the inner region of the cotyledons, it appears possible that RPK2 may act as a delimitation mechanism preventing the inadequate expansion of veins to the cotyledon margins. However, we agree with the reviewer that further experiments will be necessary to support this hypothesis. Thus, we have changed the text of this part to convey our messages (and hypothesis) more precisely. In particular, we wrote:

"The expression of *RPK2* in the vein-free cotyledon margins makes it tempting to speculate that *RPK2* may repress vein branching in a non-cell autonomous manner. "

11) Line 253: 'excluding' -> 'arguing against' I don't think an altered genetic program of mesophyll cells is completely excluded here.

This has been changed in the text and it appears in red font.

12) Line 340-341: I think to be able to make this statement, a *cvp2;cvl1;ops* triple mutant is required. Here, I would expect an enhancement of the gap phenotype.

© 2022. Published by The Company of Biologists under the terms of the Creative Commons Attribution License (https://creativecommons.org/licenses/by/4.0/).

To strengthen our claims, we have included in the current version of the manuscript and additional supplemental figure (Fig. S6) in which we are showing the branching defects in *ops* as well as in the *cvp2 ops*. As mentioned in our first comment, by using the milder vascular phenotype of *cvp2* single mutant (which exhibits mostly vascular discontinuities in the tip of the cotyledon and a reduction in proximal branching) and crossing it with *ops*, we can better assess the contribution of each gene in the regulation of this process. In particular, we observed that the reduced proximal branching observed in *ops* is enhanced in *ops cvp2*, mimicking *cvp2 cvl1*. While further experiments are required to withdraw more conclusions, it appears that *OPS* acts downstream *CVP2* and *CVL1*, consistent with previous finding performed in roots (Rodriguez-Villalon et al., Development 2015).

Reviewer #2

In the manuscript by Kastanaki and co-workers, the authors describe a logical set of experiments to understand the molecular players involved in controlling secondary vein formation in Arabidopsis embryonic leaves. Despite the importance of vascular development, most efforts are focused on root tissues; likely due to the more predictable organisation and division patterns. This makes the insights described in this manuscript important as they add to the very limited molecular knowledge about this process during leaf development. In this aspect, I believe this manuscript can be of interest to a larger group of scientists. Although I agree with the general conclusions of this manuscript which is well written; there are some aspects where the author's input seems required. These are listed below in chronological order.

1) Line18: what are plate meristematic cells? Can this be defined in the text for the non-expert readers?

Botanist Katherine Esau described in her textbook Plant Anatomy that a series of organogenesis steps in the leaf primordium depends on several distinct meristematic tissues including the plate meristem and the marginal meristem (Esau, 1977). The plate meristem consists of parallel layers of cells dividing anticlinally to play a major role in leaf growth. This terminology has been used by other authors (please see references in the text). We have slightly changed the text to introduce the concept, but due to the space limits we are confronting, we decided to keep it very simply. Please find the changes of the text in red font.

"A pair of secondary veins diverge from the midvein and extend toward the cotyledon margins as this organ expands laterally due to the proliferation of the anticlinally dividing plate meristematic cells".

2) Line92: perhaps a semantics discussion, but I thought that procambium cells in the early embryo are called pre- procambium?

We have changed it in the main text following reviewer's suggestion.

3) Line96: is there a quantification to identify these elongated narrow cells (cells are 2x as long and narrow, for example, comparing to mesophyll cells), or this is rather determined visually?

Due to the plasticity of these cells, and the difficulty imposed by working with embryos, we were not able to quantify these cells. Thus, our claims are based on our visual observations.

4) Line116 and Fig 1S-U: number of samples is not indicated in the figure nor the legend. Related, it would be better to use box plots with indication of the datapoints instead of bar charts which give limited information. This should be done throughout the manuscript. Panel U does not even have an Y- axis or error bars. The authors could also consider to homogenise the graph layouts throughout as the three in Figure 1 already are all different in size, layout etc. Related to this and more toward small details, the arrangement of the figure panels is messy with different spaces between the panels etc. Although not at all relevant to the science in this manuscript, it does give a bit of a messy appearance
In the current version of the manuscript, we have included the n number in all the panels where it was needed and have performed all esthetic corrections to improve our work as much as possible.

© 2022. Published by The Company of Biologists under the terms of the Creative Commons Attribution License (https://creativecommons.org/licenses/by/4.0/).

5) Line125: The intact midvein in the double mutant is not very clearly observed from Fig2B. Perhaps a higher magnification and quantification would help?

The midvein is formed during embryogenesis in a developmental stage that precedes the branching of secondary veins. As it can be clearly perceived in the Figure 2E, *cvp2 cvl1* midvein is always continuous (100%). This translates into a post-embryonic continuous midvein (represented in Fig. 2B). The intact midvein of *cvp2 cvl1* was previously reported (Carland and Nelson, Plant Journal, 2009). Thus, we preferred to focus in the thus-far uncharacterized phenotype of *cvp2 cvl1* which is defects in vascular branching.

6) Fig2E: the indication of 1/30 is confusing as it would suggest only 1/30 looks like WT. I assume the authors mean that 29/30 look normal in WT?
We have changed Fig. 2E and marked with numbers the frequency by which we observed the displayed phenotype.

7) Line151: please quantify the phenotype A and B in Fig S3. In panel B, what is n? Please indicate numbers of samples throughout all figure panels. We have included now the sample number in the figure.

8) Line 175: it does appear mostly basal, however I would not call PIN1 localisation in the mutant completely normal, as it seems that there is a higher amount of intracellular PIN1 aggregation. Can the authors explain what this might mean? Perhaps it would also be interesting to compare plasma membrane/intracellular signal intensity.
In *cvp2 cvl1* cells, the trafficking from the plasma membrane to the vacuole is enhanced, resulting in more TGN- dependent vesicles. While this has been reported in a previous publication (Gujas et al., Development 2017), we agree with the reviewer that is worth mentioning the differences in the overall PIN1 distribution that we observed in the mutant, even if the basal polarization remains unaffected.

"Yet, we could observe PIN1 aggregates in the intracellular space of *cvp2 cvl1* embryos, most likely due to the enhanced trafficking from the plasma membrane to vacuoles that has been previously associated to this genetic background."

9) Fig3: no statistics in the graphs, no indication of number of samples etc. Please indicate this information throughout. Panels M, N: labels are not clear for the last two bars. Are we seeing PIN1-PIN1-GFP?
We have added the information that was missing related to number of samples and performed statistical analysis. As for the legends in M and N, yes, we are characterizing transgenic lines harboring the PIN1-GFP transgene in a wild- type background (labelled as PIN1-GFP) or in *cvp2 cvl1* (labelled as PIN1-GFP *cvp2 cvl1*).

10) Line 188: both *pin1*-3 and *pin1*-5 are in the Ler background. It is interesting that Ler background lines behave so differently concerning their branching frequency in comparison to Col-0 lines. Why would this be?
We do agree with this reviewer that is surprising to observe that Ler behaves slightly different than Col. That's why we believe is important to include both genetic backgrounds as controls. In *Chen et al., Plant Phys 2013*, it was described that *ERECTA* genes regulate auxin transport in shoot apical meristem and leaf primordia. However, analysis of the vascular patterning and PIN1 distribution in the first and second leaves of the triple mutant *er erl1 erl2* did not show any differences in comparison with wild type plants. Thus, it may be possible that ERECTA may converge auxin cues that modulate cotyledon veins in a thus-far undescribed fashion. While we rate this finding as very interesting, we believe that goes beyond the scope of this paper.

11) Line196: increasing PIN1 dosage to see effect would be clearer with a 35S/overexpression promoter fusion.
We agree with the reviewer that in our transgenic plants the induction of PIN1 is very limited. However, analysis of PIN1 expressed under 35S promoter may present several caveats, as: i) 35S promoter is not expressed during embryogenesis, the developmental stage that is the main focus of this work; ii) increasing *PIN1* expression in cells where normally PIN1 is not expressed may generate an impaired auxin distribution, which in turns will impact on cotyledon vein development. Thus, we

© 2022. Published by The Company of Biologists under the terms of the Creative Commons Attribution License (https://creativecommons.org/licenses/by/4.0/).

believe that despite the limited increase observed in PIN1-GFP transgenic lines, these findings together with our genetic analysis support our claims.

12) Line 199: what phenotype would a higher order mutant (lacking functional PIN1, CVP2, CVP3) exhibit? A cross with the auxin mutants would shed light on the role in distal branching formation in the *cvp2 cvl1* double mutant.
We have generated a transgenic line harboring an artificial microRNA against PIN1 (*amiPIN1*) in *cvp2 cvl1* background. While silencing of *PIN1* expression in wild type plants slightly increase distal branching (consistent with the phenotype that we observed in *pin* mutants), the introgression of *amiPIN1*in *cvp2 cvl1* did not restore vein branching in *cvp2 cvl1*. We have included these findings in Figure 4N-T. Thus, it appears that the phenotypes of proximal branching and vein discontinuity in *cvp2 cvl1* are independent of PIN1 function.

13) Line229: quantify 'occasionally'.

The low fertility of *rpk2* plants makes the quantification of this event very challenging. However, we have included in the current version of the manuscript the quantification that we observed despite the low number of samples.

14) Line236: RPK2 involvement would be clear if ectopic expression would be observed in the *cvp2cvl1* double mutant at the branching points. The authors should provide additional explanations or hypotheses on how RPK2 can influence branch points non-cell autonomously, specifically if auxin is not involved as shown in the next paragraph.

One potential explanation for the non-cell autonomous role of RPK2 in vein branching could be through its potential activity as a co-receptor. In an exhaustive characterization of the extra-cellular LRR domains of all LRR-RK domains (Smakowska-Luzan et al., Nature 2018), RPK2 has shown to the ability to bind the brassinosteroid receptor BRI1.

Brassinosteroids have been recently described as acting non-cell autonomously in the roots and govern not only vascular development but also many other aspects of plant development (Graeff et al., Curr Biol 2020). Interestingly, BRI LIKE RECEPTOR 2 (BRL2) has been shown to interact with the vascular specific adaptor proteins VIT and VIK to modulate leaf venation (Ceserani et al., Plant Journal 2009). An increased complexity of the vascular network, which reflects higher number of branching events, can be observed in *vit* and *vik* mutants. Thus, we speculate that RPK2 may modulate BR response during embryogenesis to spatially modulate vascular development. Additionally, we have changed the discussion as follows:

"Alternatively, RPK2 may modulate the perception or response of other non-cell autonomous hormones such as brassinosteroids, whose receptors have been involved in the regulation of leaf venation. In particular, BRI1-LIKE2 appears to modulate cotyledon vein patterns through its interaction with vascular-specific adaptor proteins VIT and VIK (Ceserani et al., 2009). While thus far RPK2 extra-cellular domains has been reported to bind only BRASSINOSTEROID INSENSITIVE 1 (Smakowska-Luzan et al., 2018), further experiments may reveal whether RPK2 acts as a hub excluding vascular cues from the cotyledon margin."

15) Line 285: Obviously the authors also realise that a polar distribution in the stele does not suggest automatically that it is also polar in the leaf veins? I feel that to say that this process is PIN1 independent one would have to show PIN1:GFP or DR5 or something in the RPK2 mutants or simply show polarity in the correct place for which conclusions are drawn. This should be possible, even with low fertile plants. Related and out of curiosity, what would be the expression pattern of RPK2 in the pin1 mutant?
We agree with the reviewer that ideally the best way to study the polar localization of PIN1 would be to analyze *rpk2* veins in embryos. We have repeatedly tried to visualize PIN1-GFP and DR5::GFP in *rpk2* mutants. As shown below, PIN1-GFP polar distribution is not affected in *rpk2*. However, due to the low number of embryos analyzed (n=2), we believe that it is better not to includethese data in the manuscript. This data agrees with (i) the normal PIN1-GFP polar distribution in *rpk2* root protophloem cells; and (ii) an unaltered response of *rpk2* to NP. Taken together, these observations confirm our claims about the unrelated effect of PIN1 and RPK2 in this process. Moreover, RPK2 and PIN1 only co-localized in the tip of the cotyledon, where the auxin maxima can

© 2022. Published by The Company of Biologists under the terms of the Creative Commons Attribution License (https://creativecommons.org/licenses/by/4.0/).

be found. Thus, we would not expect to see an alteration of *RPK2* expression in *pin1* mutants.

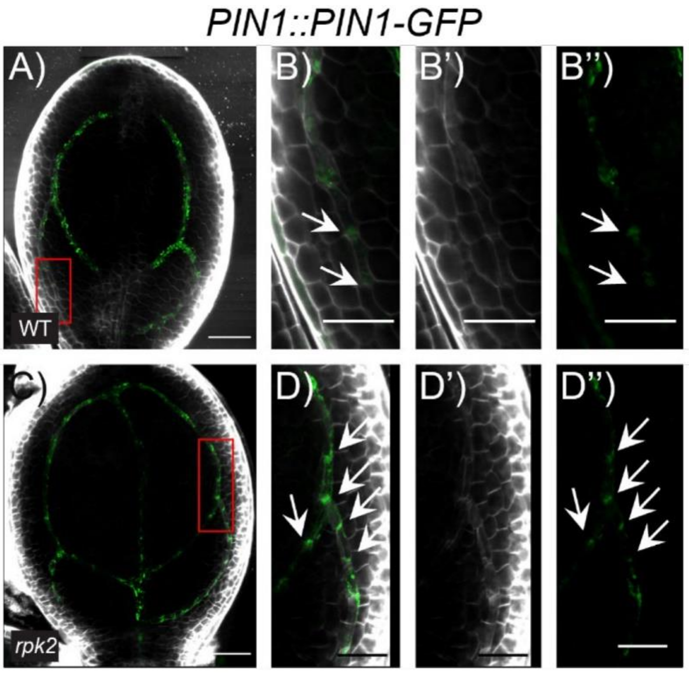

Extra Fig. 1. *rpk2* mutation does not seem to affect PIN1 polar distribution in embryonic cotyledons. Confocal microscopy analysis of WT (A-B'') and *rpk2* (C-D'') embryonic cotyledons stained with SR2200. Due to the low fertility of *rpk2* plants, only 2 cotyledons from mature embryos were analyzed. Magnifications of the regions squared in red in A (B-B'') and C (D-D'') are displayed. Scale bars represent 50µm in (A,C) and 25µm in (B-B'', D-D''). White arrows show the PIN1 polar distribution in the corresponding vascular cells.

16) Line333: could RPK2 and OPS interaction perhaps at the sites of overlapping expression? What would the implication be?
Please see the answer to comment 14. RPK2 has been reported to interact with BRI1, but not interaction with OPS has been described thus far. Yet, and as we comment in our discussion, further experiments assessing the potential interaction between RPK2 and OPS proteins may help to elucidate whether both proteins can interact or whether they regulate this process by two independent pathways. However, we believe that this characterization goes beyond the scope of the current manuscript.

17) Line 336: does the ops mutant show a defect in branching? The phenotype of *ops* in terms of vein branching has been included in the current manuscript in the supplemental figure 6. In particular, we observed that *ops* cotyledons display a reduced number of proximal branching points, confirming its role as a positive regulator of this process.

18) Line 341: Do higher order mutant combinations between OPS, RPK2, CVP2 or CVL1 exist? If so, it would be good to see whether they are in the same pathway/epistatic/additive/etc. I can however imagine that it would be difficult to get these higher order mutants due to lethality.
We do agree that the analysis of *cvp2 cvl1 ops* would be interesting for the paper. In this triple mutant we would expect to see an equally impaired or even more severe cotyledon vascular network than in *cvp2 cvl1*. Unfortunately, we were not able to generate neither *cvp2 cvl1 ops* nor *rpk2 ops* to determine the contribution of each genes in the regulation of this process. Hence, we have decided to take advantage of *cvp2* single mutants (described in Rodriguez- Villalon et al., Development 2015), which shows a milder defective vascular phenotype than *cvp2 cvl1* in terms of branching, and we crossed it with *ops*. While *cvp2* exhibits defects in proximal branching, this phenotype is more severe when additionally knocking out the activity of *ops*, mimicking *cvp2 cvl1*. This result is consistent with the notion of OPS acting downstream CVP2/CVL1 that has been reported in the context of root phloem regulation (Rodriguez- Villalon et al., 2015). However, it doesn't argue against an additive role. Further experiments would be needed to precisely

© 2022. Published by The Company of Biologists under the terms of the Creative Commons Attribution License (https://creativecommons.org/licenses/by/4.0/).

determine the weight of each gene in the regulation of this process. Analysis of the *cvp2 ops* double mutant is now displayed in the new Supplemental Figure 6. The description of its phenotype appears in the text as follows:

"Consistent with these findings, the mild branching phenotype observed in *cvp2* single mutants was more pronounced when introgressed in *ops* genetic background (Fig. S6A-C)."

19) Line399: according to Sup Fig 1, introduction of PIN1:PIN1-GFP hardly increased (not even two-fold, in fact its comparable to control PIN1-GFP line) dosage in the mutant. Perhaps using a different promoter would be more useful, otherwise it can be hard to claim that it does not enhance branching based on what is shown here. Please see the answer to comment 11.

Second decision letter

MS ID#: DEVELOP/2021/200403

MS TITLE: A genetic framework for proximal secondary vein branching in the *Arabidopsis thaliana* embryo.

AUTHORS: Elizabeth Kastanaki, Noel Blanco-Touriñán, Alexis Sarazin, Alessandra Sturchler, Bojan Gujas, Francisco Vera-Sirera, Javier Agustí and Antia Rodriguez-Villalon
ARTICLE TYPE: Research Article

I am happy to tell you that your manuscript has been accepted for publication in Development, pending our standard ethics checks.

Reviewer 1

Advance summary and potential significance to field

I rate this study as being very well conducted, the amount of accumulated data is immense and I do have hardly any concern with regard to the experimental strategy or data generation/presentation. The question toward the formation of plastic vein patterning in plant leaves, is certainly fundamental and served as a paradigm for pattern formation in the past. Moreover, performing these analyses during embryogenesis is technically challenging and has (as far as I am aware) not been performed before. Therefore, the study allows insights into the process with an unprecedented depth and amount of information. In this regard, the authors make efficient use of their analytical skills and of existing technical and genetic resources.

Comments for the author

In the revised version of the manuscript, the authors have addressed all my concerns and I do not have anything to add or suggest anymore at this stage.

Reviewer 2

Advance summary and potential significance to field

In this revised manuscript, the authors still describe a logical set of experiments to understand the molecular players involved in controlling secondary vein formation in Arabidopsis embryonic leaves. Despite the importance of vascular development, most efforts are focussed on root tissues; likely due to the more predictable organisation and division patterns. This makes the insights described in this manuscript important as they add to the very limited molecular knowledge about this process during leaf development. In this aspect, I believe this manuscript can be of interest to a larger group of scientists.

© 2022. Published by The Company of Biologists under the terms of the Creative Commons Attribution License (https://creativecommons.org/licenses/by/4.0/).

Comments for the author

The authors have adequately addressed all comments and I thus have no further remarks.

© 2022. Published by The Company of Biologists under the terms of the Creative Commons Attribution License (https://creativecommons.org/licenses/by/4.0/).
